# Fragment length profiles of cancer mutations enhance detection of circulating tumor DNA in patients with early-stage hepatocellular carcinoma

**DOI:** 10.1186/s12885-023-10681-0

**Published:** 2023-03-13

**Authors:** Van-Chu Nguyen, Trong Hieu Nguyen, Thanh Hai Phan, Thanh-Huong Thi Tran, Thu Thuy Thi Pham, Tan Dat Ho, Hue Hanh Thi Nguyen, Minh-Long Duong, Cao Minh Nguyen, Que-Tran Bui Nguyen, Hoai-Phuong Thi Bach, Van-Vu Kim, The-Anh Pham, Bao Toan Nguyen, Thanh Nhan Vo Nguyen, Le Anh Khoa Huynh, Vu Uyen Tran, Thuy Thi Thu Tran, Thanh Dang Nguyen, Dung Thai Bieu Phu, Boi Hoan Huu Phan, Quynh-Tho Thi Nguyen, Dinh-Kiet Truong, Thanh-Thuy Thi Do, Hoai-Nghia Nguyen, Minh-Duy Phan, Hoa Giang, Le Son Tran

**Affiliations:** 1 National Cancer Hospital, Hanoi, Vietnam; 2 Hanoi Medical University, Hanoi, Vietnam; 3 Medical Genetics Institute, 186 Nguyen Duy Duong, Ward 3, District 10, Ho Chi Minh City, Vietnam; 4 Gene Solutions, Ho Chi Minh City, Vietnam; 5 MEDIC Medical Center, Ho Chi Minh City, Vietnam; 6 Virginia Commonwealth University, Richmond, USA; 7 University of Medicine and Pharmacy at Ho Chi Minh City, Ho Chi Minh City, Vietnam

**Keywords:** Hepatocellular carcinoma, Cell free DNA, Circulating tumor DNA, Liquid biopsy, Cancer specific mutation, Fragmentomics, Target enrichment, Variant calling, Gradient boosting model

## Abstract

**Background:**

Late detection of hepatocellular carcinoma (HCC) results in an overall 5-year survival rate of less than 16%. Liquid biopsy (LB) assays based on detecting circulating tumor DNA (ctDNA) might provide an opportunity to detect HCC early noninvasively. Increasing evidence indicates that ctDNA detection using mutation-based assays is significantly challenged by the abundance of white blood cell-derived mutations, non-tumor tissue-derived somatic mutations in plasma, and the mutational tumor heterogeneity.

**Methods:**

Here, we employed concurrent analysis of cancer-related mutations, and their fragment length profiles to differentiate mutations from different sources. To distinguish persons with HCC (PwHCC) from healthy participants, we built a classification model using three fragmentomic features of ctDNA through deep sequencing of thirteen genes associated with HCC.

**Results:**

Our model achieved an area under the curve (AUC) of 0.88, a sensitivity of 89%, and a specificity of 82% in the discovery cohort consisting of 55 PwHCC and 55 healthy participants. In an independent validation cohort of 54 PwHCC and 53 healthy participants, the established model achieved comparable classification performance with an AUC of 0.86 and yielded a sensitivity and specificity of 81%.

**Conclusions:**

Our study provides a rationale for subsequent clinical evaluation of our assay performance in a large-scale prospective study.

**Supplementary Information:**

The online version contains supplementary material available at 10.1186/s12885-023-10681-0.

## Introduction

Primary liver cancer was the sixth most diagnosed cancer and the third leading cause of cancer death worldwide in 2020, with approximately 906,000 new cases and 830,000 deaths. Incidence rates are the highest in transitioning countries, with the disease being the most common cancer in 11 geographically diverse countries [1]. More than 60% of patients are diagnosed with late-stage disease after metastasis has occurred, resulting in an overall 5-year survival rate of < 16% [2]. Hepatocellular carcinoma (HCC) encompasses 75%-85% of primary liver cancers. Early-stage HCC is potentially responsive to curative treatment, ranging from local ablation to liver transplantation [3]. Early detection improves patients' survival rates: patients diagnosed with early-stage disease have a relatively good prognosis, with a 5-year survival rate of > 70% [4]. Specifically, in patients diagnosed with early-stage HCC, such as Barcelona Clinic Liver Cancer stage 0 and A, the 5-year survival rate with the surgical intervention was > 93% [4]. The improvement of early detection is vital for low-resource settings where HCC often develops early without pre-existing cirrhosis, thus removing the early sign of a risk factor [3].

Current diagnostic tests based on serum protein biomarkers give high false-positive results, and despite improvements, early cancer detection continues to face challenges [5, 6]. Liquid biopsy (LB) assays based on the detection of circulating tumor DNA (ctDNA) have recently emerged as noninvasive and accessible tools for the early detection of multiple cancer types [7–10]. ctDNA accounts for a small proportion of circulating cell-free DNA (cfDNA) in the blood and can be distinguished from benign cfDNA by specific markers such as mutations in genes known to be cancer-related [7, 11, 12]. There are several cancer-related mutations that allow ctDNA to be distinguished from cfDNA. CancerSEEK, a multi-analyte blood test, was used to survey 1,005 participants with clinically detected non-metastatic forms of one of eight common cancer types (breast, colorectal, esophageal, liver, lung, ovarian, pancreatic, and stomach). Evaluating levels of eight proteins and the presence of mutations in 1933 distinct genomic positions, a positive CancerSEEK test was classified as the presence of a mutation in an assayed gene or an elevated level of any of the proteins. The tests had a median sensitivity of 70% (ranging from 69 to 98%) for detecting these eight cancer types [7]. However, there are several characteristics of cfDNA that hamper its use as a diagnostic tool. First, the extremely low concentration of tumor-derived cfDNA found in a blood draw reduces the ability to detect early-stage tumors [6]. Additionally, some patients have low ctDNA even during late-stage disease [8]. This low proportion, coupled with the low variant allele frequency (VAF) found in somatic mutations in tumor-derived cfDNA, causes problems for traditional single nucleotide variant (SNV) callers [9]. Second, because cfDNA contains multiple sources of DNA, hematopoiesis mutations formed from the clonal proliferation of blood cells can lead to false-positive findings and confound LB interpretation [13, 14]. This high contribution of cfDNA with a wide range of somatic mutations creates a bias [14]. Third, somatic mosaicism, or normal cells carrying benign somatic mutations, is common in healthy people across many organs and tissues. Somatically mutated DNA enters the blood-lymph system and contributes to the circulating cfDNA [15].

In addition to mutations, features of the cfDNA fragments, like size, single-stranded jagged ends, and endpoint locations, have also been exploited to develop noninvasive screening and diagnostic assays [16]. An early study of plasma cfDNA found varying fragment sizes between benign adnexal masses and malignant gynecological neoplasms [17]. While cfDNA of participants with hepatitis B virus (HBV) infection, cirrhosis, and HCC contained fragments sized at an average of about 166 bp, the plasma DNA of cancer patients had both shorter and longer fragment distribution [18]. In contrast to cfDNA from healthy people, cancer patients had numerous distinct genomic differences, including longer and shorter fragments at different regions [19]. Several studies have shown that cfDNA fragments harboring mutant alleles were often shorter than those with wild-type alleles [20–22]. Size selecting for shorter cfDNA fragments increases the proportion of ctDNA within a sample [21]. For example, the cfDNA of a group of lung cancer patients was more fragmented than that of healthy controls, with an average length of 134 to 144 bp. Thus, tracking the mutational landscape and fragmentation of plasma cfDNA might have promising diagnostic potential [20].

This study addressed the challenges of using cancer-associated mutations to detect ctDNA in a discovery cohort of 55 patients with early-stage HCC and 55 healthy individuals. To overcome these challenges, we developed an assay based on the aggregation of fragment length profiles of mutations in the 13 most frequently mutated genes associated with HCC. We evaluated the performance of our assay in both the discovery cohort and an independent validation cohort of 54 PwHCC and 53 healthy participants from a different hospital.

## Materials and methods

### Study design and patient enrollment

In the discovery cohort, a total of 55 patients with HCC confirmed by imaging diagnosis and histopathological analysis and 55 healthy participants from the National Cancer Hospital, Hanoi, Vietnam, were recruited to this study from March 2019 to December 2021 (Fig. S1). The recruitment criteria for HCC patients were early stage (stage I, II) or with non-metastatic disease (stage IIIA) and naive to treatment. Healthy participants had no diagnosis of cancer or previous history of cancer.

In the validation cohort, our study included 54 patients with non-metastatic HCC plus 53 healthy individuals from the Medic Medical Center, Ho Chi Minh City, Vietnam, recruited from July 2019 to December 2021 to validate the performance of our assay (Fig. S1). HCC patients in the validation cohort visited the Medic Medical Center for diagnostic imaging examinations but went to other hospitals for treatment. Therefore, their tumor tissue samples were not available for histopathological analysis.

Written informed consent was obtained from all patients for tumor and whole blood samples. Written consent was obtained from all healthy controls. Clinical data (demographics, cancer stages, and pathology data) was collected from medical records at the National Cancer Hospital. Comprehensive details of patients' clinical factors are summarized in Tables S1A and B.

This study was approved by the Ethics Committee of the National Cancer Hospital and Medic Medical Center.

### Whole blood and tumor sample processing

Liquid biopsy, and white blood cell samples were taken from all 55 PwHCC in the discovery cohort. Of those patients, 44 had available tissue biopsies. Only blood samples were collected from 54 PwHCC in the validation cohort. Additionally, blood samples were collected from all 108 healthy participants, including 55 in the discovery cohort and 53 in the validation cohort.

Ultra-deep targeted sequencing was used to determine the sequence of the 13 genes most frequently associated with HCC according to the COSMIC database [23]. Peripheral blood samples were collected in 10-ml Streck tubes (Cell-free DNA BCT, Streck) and stored at room temperature for a maximum of 8 h before undergoing plasma isolation. Whole blood was separated into plasma and buffy coat via centrifugation (2000 × g for 10 min and 16,000 × g for 10 min) and stored at -80º C and -20º C, respectively. cfDNA was extracted from 1 ml of plasma using the MagMAX cell-free DNA Isolation Kit (Thermo Fisher, USA) following the manufacturer's instruction.

From patients with HCC, formalin-fixed, paraffin-embedded (FFPE)-tumor samples and plasma with matched white blood cell (WBC) DNA from the peripheral blood were collected. Tumor DNA and WBC DNA from matching buffy coat were isolated using the QIAGEN FFPE DNA Mini Kit (QIAGEN, USA) and the MagMAX DNA Multi-Sample Ultra 2.0 Kit with KingFisher Flex automated instrument (Thermo Fisher, USA), respectively. Genomic DNA (gDNA) was fragmented with NEBNext dsDNA Fragmentase (New England Biolabs, USA) at 37º C for 15 min. The reaction was stopped immediately with 0.5 M EDTA. Sheared gDNA was selected by size (100–1000 base pairs) using KAPA Pure Beads (Roche, Switzerland). DNA concentration was measured using the QuantiFluor dsDNA system (Promega, USA).

### Library preparation, target enrichment, and sequencing

Sheared gDNA (30 ng) and plasma cfDNA (≥ 1.5 ng) were used for NGS library construction with the ThruPLEX Tag-seq Kit (Takara Bio, USA). The stem-loop adapters provide 16 million unique molecular tags (UMT) to reduce the technical assay error rates and ensure the correct base call throughout data analysis.

DNA sequencing was performed using the MGI DNBSEQ Sequencing Technology (BGI, China) with 100 paired-end read lengths for a total of 222 cycles per the manufacturer's guidelines. Quantified libraries were enriched with a self-built panel for targeted genes. The panel comprised 12 genes with high mutation frequencies in HCC and a TERT-promoter region that correlated with an increased risk of HCC (Table S2) [24]. Up to 1500 ng of cfDNA and gDNA libraries were used for hybridization capture with IDT xGen Lockdown Reagents (IDT, USA). We designed a xGen Lockdown probe panel spanning the entire coding regions of the 12 selected genes and the promoter region of *TERT* (Table S2). Thus, multiple mutations within these regions were called and summarized in Table S3. Hybridization performance was analyzed by target rate and percentage of reads mapped.

### Data analysis and variant calling pipeline

#### Variant calling pipeline

The data analysis and variant calling pipeline began with trimming the raw sequencing reads in demultiplexed FASTQ from 100 to 75 bp by *Trimmomatic v.0.39* from the 3' end [25]. For quality control of FASTQ files, we ran *fastqc v.0.11.8.* We implemented a custom pipeline to process the Unique Molecular Identifier (UMI) following the trimming process. We extracted and assigned UMI to all reads starting with an alignment process (*BWA-MEM v.0.7.17*) of raw reads to the human reference genome (*hg38*) [26]. Reads were then grouped by their UMI, followed by calling consensus sequences from reads with the same UMI. These processes are performed with the help of *fgbio* package *v.1.4.0* [27, 28]*.* Finally*,* we performed variant calling with *Vardict-Java v.1.5.1* after aligning all consensus reads to the human reference genome (*hg38*) [29]. Note that we only used the single-sample mode of Vardict; our custom framework filtered called variants*.* Variant annotations were queried by *VEP v.99* from *COSMIC (v.94)*^23^ and *Clinvar* [30]*.* For processing aligned reads in SAM/BAM format, we use *samtools v.1.14* [31]*, bedtools v.2.30* [32], and *picard v.2.18.7* [33]*.* All variants were examined but only those with VAF less than 0.1 were included in the construction of fragmentomic features. We also developed our in-house *Python* scripts for the analysis, including the following packages: *pandas, numpy, scipy, matplotlib, seaborn, scikit-learn, xgboost, pyoncoprint* [34]*.*

#### Fragment length analysis

For fragment length (Flen) distributions, we implemented an in-house python script to convert the bsalign files into BAM files. All read pairs from the BAM file with fragment length ranging from 100 to 250 bp were collected. In the range of 100 to 250 bp, there were 151 possible fragment lengths, starting from length of 100 bp, with 1 bp increment, up to 250 bp. With each length, the frequency of fragment (%) was calculated by getting the percentage of reads with each length to the total read count in the range of 100 to 250 bp. This calculation resulted in a feature vector of 151 dimensions. We plotted fragment length (bp) against frequency of fragment (%) to obtain a Flen distribution curve.

For ratio of short fragment to long fragments, the whole genome was segmented into non-overlapping bins of 5 Mb (5 million bases). Bins from chromosomes X and Y were excluded in this analysis. Reads which were shorter than 100 or longer than 250 bp were removed. In each bin, short reads, whose read lengths were from 100 to 150 bp, and long read, whose read lengths were from 151 to 250 bp were collected. The ratio of the number of short reads to the number of long reads in each bin was calculated. This finally resulted in a feature vector of 588 dimensions corresponding to 588 bins of 5 Mb.

#### Fragmentomic features

We followed an approach inspired by Chabon et al. [35] to build a classification model using fragmentomic features. First, we employed a statistical model to eliminate potential WBC variants that could also exist in LB samples. Variants from a cohort of 55 WBC samples of cancer patients and 55 WBC samples of healthy-control individuals were pooled together. We adopted the construction of a WBC Bayesian background model [35] for this purpose: We modeled a background distribution and a zero-inflated beta distribution for the WBC variants coming from these 110 samples based on their VAF, allele depth (AD), and total read depth (DP). For a given variant found in a LB sample and the WBC cohort, we measured the difference between the variant and the background distribution. A *p*-value emphasizing the significance of the difference was calculated. Only variants having *p*-value less than 0.05 were kept and denoted by LB unique. It is expected that, by this method, variants whose profiles demonstrate a similar pattern in VAF to WBC variants were excluded. Finally, we constructed the following features using these selected variants:

- Feature 1: Fraction of short-to-long Alternate (ALT)-fragments

For each mutation in the LB-unique set, we fetched all reads overlapping its genomic coordinates and calculated their fragment lengths. Fragments having the mutation of interest were denoted ALT-fragments, and the remaining fragments were labeled REF (reference)-fragments.

We constructed the first fragmentomic feature as the fraction of short ALT-fragments (ALT-fragments shorter than 150 bp) to long ALT-fragments (ALT-fragments longer than 150 bp) with all mutations (Fig. S5A). Since tumor-derived fragments would be more likely to be shorter than normal fragments [36], this feature could be considered as a measure for abundance of tumor-originated fragments in cancer patients.

- Feature 2: Size selection enrichment test

Fragment length distribution of all fragments overlapping LB-unique mutations in cancer patients differs from that of the healthy individuals (Fig. S5B). However, these differences are only significant in some specific ranges regarding the number of ALT and REF-fragments (Fig. S5B). A Fisher's exact test for the following contingency table succinctly describes this observation.

**Table:** 

	$110 \le s \le 135$	$s < 110$ or $s > 135$
Number of REF-fragments	A	B
Number of ALT-fragments	C	D

The Phil's read editor (PHRED)-scaled *p*-value obtained from this test will be the second feature in our machine learning model.

- Feature 3: The probability of observing a fragment of size s in ALT-fragments and REF-fragments

Roughly speaking, we computed the probability of observing an ALT-fragment of size $s$, for $s\in \{100, 101,..., 250\}$ in all ALT fragments by proportions (Fig. S5C)

$$Prob(f=s\vert f\in ALT\;fragments)=\frac{\#\{number\;of\;ALT\;fragments\;of\;size\;s\}}{\#\{total\;number\;of\;ALT\;fragments\}}$$

Similarly,

$$Prob(f=s\vert f\in REF\;fragments)=\frac{\#\{number\;of\;REF\;fragments\;of\;size\;s\}}{\#\{total\;number\;of\;REF\;fragments\}}$$

We then calculate

$$\lambda(s):=\frac{Prob(f=s\vert f\in ALT\;fragments)}{Prob(f=s\vert f\in REF\;fragments)}$$

The third feature will be the sum of $\lambda (s)$ over a sliding and non-overlapping window of 10 consecutive values of $s$, $s\in \{100, 101,..., 250\}$, which yields a 15-dimensional feature vector.

### Machine learning model and training procedure

#### Machine learning model

We trained a gradient-boosting tree-based algorithm to classify cancer patients versus healthy individuals. The set of features used in this model combined all three features described previously, generating a data matrix in ${R}^{N \times 17}$, where $N$ denotes the number of samples. Parameter tuning was performed using a grid-search strategy.

#### Leave-one-out cross validation

We split the dataset into a discovery and validation cohort. The discovery cohort has 55 PwHCC and 55 healthy participants. We trained the model with the leave-one-out cross-validation procedure to examine the model's performance. The overall results, sensitivity and specificity, and the optimal threshold for the base score of predicted probability were determined by the Receiver Operating Characteristic (ROC) analysis and Youden's index [37].

The external validation comprised of 54 blood samples taken from PwHCC from a different hospital than the discovery cohort’s, along with 53 healthy participants. This cohort served as an independent validation set for our trained machine learning model (Fig. S1).

### Statistical analysis

The Wilcoxon signed rank test was used to compare the median age and differences in fragmentomic features of HCC patients and healthy subjects. Chi-squared (*χ*2) test was performed to compare gender ratios between HCC and healthy controls. Pearson's correlation coefficient test was used to assess correlations between WBC-derived mutational VAFs in LB and WBC samples. All statistical analyses were carried out using Python (v3.7) with some common data analysis packages: *numpy, scipy, pandas*.

## Results

### Clinical characteristics of HCC patients and healthy participants in discovery and validation cohorts

For the discovery cohort, we collected blood and tumor samples from 55 patients with stage I, II, and IIIA HCC and blood samples from 55 healthy participants from the National Cancer Hospital in Hanoi (Table 1, Fig. S1). Most of the patients were men (85.5%), and the vast majority of the patients had hepatitis B infection (78.2%). Patients with stages I and II account for most patients (Stage I: 16.4%; stage II: 52.7%). To validate our assay, we additionally recruited 54 HCC patients and 53 healthy individuals from another hospital, the Medic Medical Center in Ho Chi Minh City (Table 1, Fig. S1). For those patients, only blood samples were collected thus histological analysis is not available. All cancer patients in the discovery cohort were confirmed to have non-metastatic cancer by imaging diagnosis and histological analysis. All cancer patients in the validation cohort were confirmed to have non-metastatic cancer by imaging diagnosis. The median age and gender ratio of cancer patients and healthy individuals in this cohort are comparable to those of the discovery cohort (Table 1). In both the discovery and validation cohort, HCC patients had significantly higher median age and male to female ratios than healthy controls.

**Table 1:** Characteristics of Discovery and Validation cohorts

	**Discovery cohort**		**Validation cohort**	
	PwHCC (*N* = 55)	Healthy participants (*N* = 55)	PwHCC (*N* = 54)	Healthy participants (*N* = 55)
	N (%)	N (%)	N (%)	N (%)
**Gender**^a^				
Female	8 (14.5)	32 (58.2)	16 (29.6)	29 (54.7)
Male	47 (85.5)	23 (41.8)	38 (70.4)	24 (45.3)
**Age**^b^				
Median	58	42	60	40
Min	24	25	33	26
Max	87	81	77	67
**Stage**				
I	9 (16.4)	NA	All patients were confirmed to have non-metastatic HCC	NA
II	29 (52.7)	NA		NA
III	3 (5.5)	NA		NA
NA	14 (25.5)	NA		NA
**Risk factor**				
HBV	43 (78.2)	NA	32	NA
HCV	3 (5.5)	NA	10	
HBV/HCV	1 (1.8)	NA	1	
No	6 (10.9)	NA	7	
Not detected	2 (3.6)	NA	3	

^a^ Chi-square values of ratio of female to male: Discovery cohort, *p* = 5.146e-06; Validation cohort, *p* = 0.015

^b^ Wilcoxon signed rank test for ages: Discovery cohort, *p* = 2.444e^−06^; Validation cohort, *p* = 4.223e^−10^

### Plasma samples of HCC patients (PwHCC) contains an abundance of WBC-derived mutations

Our previous study and others have shown that plasma cfDNA and tumor tissues contain mutations from noncancerous blood cells and cancer cells [14, 38]. Thus, one major challenge to using tumor-derived mutations (TDM) as biomarkers for detecting ctDNA in plasma is that many characteristics overlap with WDM. To address this challenge in PwHCC, we sequenced plasma cfDNA and paired white blood cell (WBC) genomic DNA from all 55 PwHCC in the discovery cohort.

Our sequencing assay examined the 12 most frequently mutated genes in HCC and promoter region of *TERT* according to the COSMIC database (Table S2). We also employed UMI technology to suppress sequencing errors. DNA sequencing data were obtained from all PwHCC with on target rates > 50%, and comparable UMI consensus read coverage ≥ 500X, for plasma cfDNA and WBC gDNA (Figs. S2A and B). By using at least 1.5 ng of cfDNA (ie. ≥ 1.5 ng, IQR: 3.74-7.15, Fig. S2C) for library preparation, we achieved sequencing depth coverages consistent with a previous study which used 1.7 to 10 ng of cfDNA and achieved mean coverage of the targeted bases of 462.6X by performing target capture for a gene panel with comparable size [39].

**Fig. 1:**
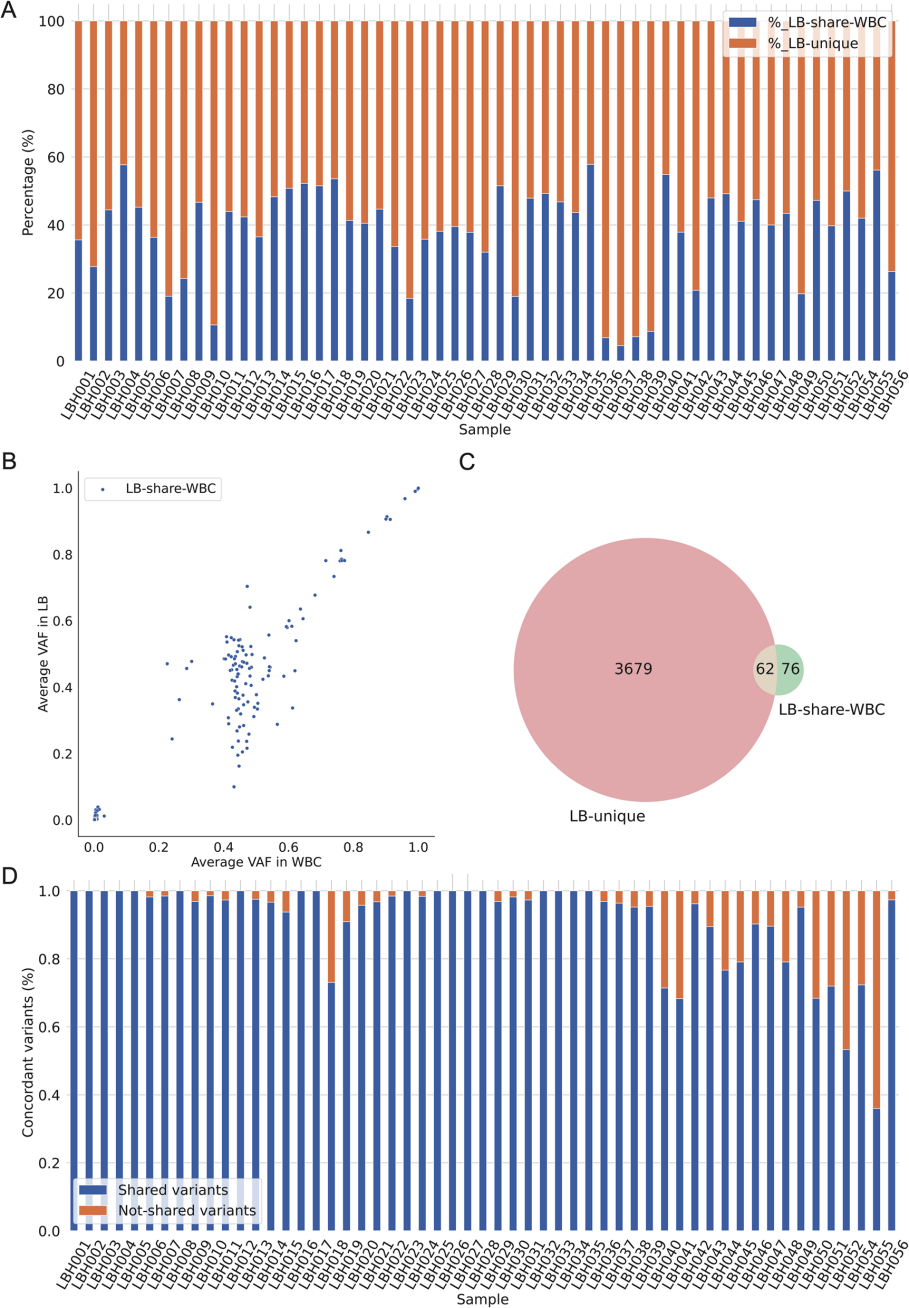
Identification of white blood cells (WBCs) derived mutations in liquid biopsies of HCC patients. **A** Detection rates of mutations shared between liquid biopsies and paired WBCs (LB-share-WBC) and mutations uniquely found in liquid biopsy (LB) samples (LB-unique) (*n* = 55). **B** Correlation of the mean VAFs of LB-share-WBC mutations in WBCs and LBs. *p*‐values and correlation coefficients (r) were calculated using Pearson's correlation test. **C** Venn diagram showing overlapping LB-share-WBC and LB-unique spectra.  (yellow) LB and WBC shared.  (pink) LB-unique.  (green) WBC unique. **D** The concordance rates of WBC-derived mutations detected by parallel sequencing of paired WBC gDNA and plasma cfDNA with in-house model

Consistent with previous findings, many of the mutations found in cfDNA fragments from the plasma of PwHCC were also found in the buffy coat portion of the plasma (LB-share-WBC), indicating that these mutations originated in the WBC (Fig. 1A). LB-share-WBC mutations accounted for about 41.3% of the mutations in plasma cfDNA, ranging from 4.5% to 57.8% of mutations (Fig. 1A, Table 2).

**Table 2:** Descriptive statistics of the proportion of LB-unique and LB-share-WBC or the discovery cohort

	#_total	#_LB-unique	#_LB-share-WBC	%_LB-unique	%_LB-share-WBC
count	55.0	55.0	55.0	55.0	55.0
mean	114.6	83.7	31.0	62.0	38.1
std	114.9	114.7	4.31	14.0	14.0
min	52.0	22.0	20.0	42.2	4.5
25%	64.5	32.0	28.5	52.0	32.8
50%	73.0	43.0	32.0	59.0	41.3
75%	96.0	60.5	34.0	67.2	47.9
max	663.0	633.0	41.0	95.5	57.8

Moreover, the VAFs of LB-share-WBC mutations in plasma highly correlated with their VAFs in WBCs (*r* = 0.95, 95% CI 0.94–0.97, *p* < 0.0001, Fig. 1B). This finding further confirmed that WDMs are the major constituents of PwHCC's LB. LB-share-WBC mutations with VAF > 0.2 are likely germline mutations, while those with VAF < 0.2 might arise from clonal hematopoiesis. After excluding LB-share-WBC mutations, the remaining non-overlapping mutations denoted as LB-unique mutations (LB-unique) might be of tumor origin (median: 58.7%, range: 42.2%–95.5%, Fig. 1A, Table 2).

Of the 138 detected WDMs, 62 (44.9%) overlapped with LB-unique detected across individual patients (Fig. 1C), indicating that the spectra of WDMs and LB-unique mutations are not distinct, or that a LB-share-WBC mutation in a particular person could be an LB-unique mutation in another. This lack of distinction suggests that parallel sequencing of cfDNA and matched WBC gDNA at equal depth is required to identify LB-share-WBC mutations in the plasma of each patient to avoid misinterpretation with mutations that are potentially of tumor origin. Since this approach is cost-prohibitive, we developed an in-house probabilistic model to distinguish LB-share-WBC mutations from LB-unique mutations in plasma by modeling the distributions of VAFs, occurrences, and allele read depth of the two mutation groups. Compared to the parallel sequencing approach, the model achieved a high concordance rate of 91.7% in identifying LB-share-WBC mutations in 55 HCC samples (Fig. 1D). Our findings showed that WBC-derived mutations are abundantly present in the plasma of PwHCC and could be accurately determined by using a probabilistic model.

### Heterogeneity and overlap of tumor-derived mutations with mutations detected in healthy individuals

**Fig. 2:**
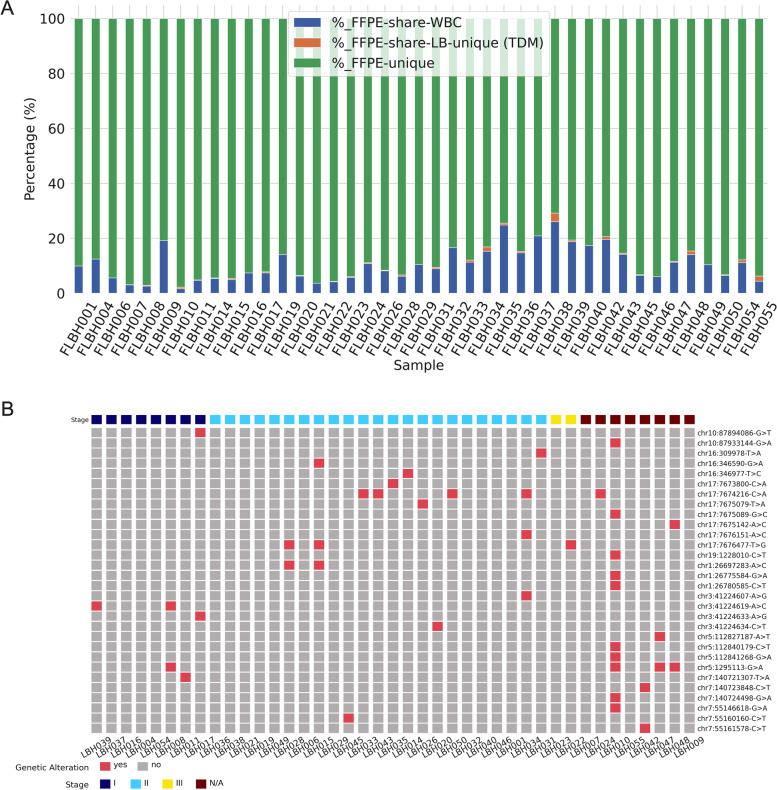
Tumor-derived mutations in plasma samples of PwHCC displayed heterogeneity. **A** Proportion of different mutation groups in tumor tissues from 55 PwHCC, including mutations overlapping WBC-mutations (FFPE-share-WBC), with mutations detected in paired plasma samples (TDMs or FFPE-share-LB unique) or those uniquely detected in tumor tissues (FFPE-unique). **B** Oncoprint plots of distributions of TDMs in 49 PwHCC with paired plasma and tumor tissues. Rows and columns represent TDMs and patients, respectively. Mutations are labeled on the right side. The left-side bar plot shows the occurrences of each mutation, among all patients, while the top most bar plot represents the mutational loads of each patient. HCC Patients are grouped according to their tumor stages

We performed sequencing on patient-paired tumor tissues to identify possible tumor origins of LB-unique fragments. Of the 55 HCC patients, only 41 underwent tissue biopsy and had available tumor tissue samples. WBC derived mutations (WDMs) were also detected in the paired tumor tissues of those patients (FFPE-share-WBC) at lower rates than LB samples, with a median of about 9.9% of the mutations (range 1.6%-26.0%, Fig. 2A, Table 3).

**Table 3:** Number and proportion of different types of mutations detected in FFPE samples

	**#_total**	**#_FFPE-unique**	**#_FFPE-share-WBC**	**#_FFPE-share-LB**	**#_FFPE-share-LB-unique (TDM)**	**%_FFPE-unique**	**%_FFPE-share-WBC**	**%_FFPE-share-LB-unique (TDM)**
**count**	41	41	41	41	41	41.0	41.0	41.0
**mean**	424	392	31	31	2	89.0	10.5	0.4
**std**	302	302	4	4	2	6.4	6.2	0.6
**min**	96	68	21	23	0	70.8	1.6	0.0
**25%**	225	193	28	28	0	84.8	5.8	0.0
**50%**	338	303	31	31	1	90.1	9.9	0.2
**75%**	539	508	33	34	2	94.0	14.2	0.6
**max**	1720	1682	39	41	10	97.8	26.0	3.1

These could be germline mutations or be derived from tumor-infiltrating lymphocytes. After excluding such mutations, only a small proportion of mutations in tumor tissues overlapped with LB-unique mutations (FFPE-share-LB-unique, median: 0.2%, range 0–3.1%, Fig. 2A, Table 3); these were denoted as tumor-derived mutations (TDMs). Thus, not all mutations detected in tumor tissues were shed into the circulation and the remaining mutations uniquely detected in tumor tissues (FFPE-unique) constituted the majority (median: 90.1%, range 70.8%- 97.8%). LB-unique mutations that were not confirmed in paired tissues were defined as variants of unknown source (VUS). Such VUS could be derived either from tumor clones that were lost during tissue sampling or from unknown sources, as previously described [14].

Of the 41 LB samples from patients with available paired tumor tissues, 22 (53.6%) had at least one TDM (Fig. 2B). TDMs were detected in all stages of HCC (Fig. 2B). Of 30 identified TDMs, two mapping to *TP53* (chr17: 7,674,216-C > A) and *TERT* promoter (chr5: 1,295,143-G > A) were shared by 5 and 4 patients, respectively. The majority (25/30, 83.3%) of TDMs were not shared among PwHCC, thus highlighting the inter-individual heterogeneity of TDMs (Fig. 2B). In addition, we found that 3/30 (10%) TMDs were found in 10/55 (18.2%) of the blood samples of healthy control participants (Fig. S3A). These shared mutations are most likely benign somatic mutations, leading to the false-positive detection of cancer patients. These findings presented the heterogeneity of TMDs detected in plasma samples of PwHCC and their overlapping profiles with benign somatic mutations in LB samples of healthy individuals, highlighting the importance of using the presence of the HCC-associated mutations as markers to distinguish PwHCC from healthy individuals.

### TDM fragments and LB-unique fragments from PwHCC display size distribution profiles distinct from the reference sequences with an increased proportion of short fragments

It has been well established that cfDNA shed by cancer cells (ctDNA) tend to be shorter than cfDNA shed by other normal cells [19, 21]. Consistent with a previous study by Jiang et al. [18], we found that short DNA fragments (< 150 bp) appeared more frequently in the plasma of HCC patients compared to healthy individuals (Fig. S4A). Moreover, liver cancer patients had markedly higher ratios of short (< 150 bp) to long (> 150 bp) cfDNA fragments across the entire genome than healthy individuals (Fig. S4B). These data provide a rationale for exploiting these signatures in combination with cancer mutations to detect HCC.

**Fig. 3:**
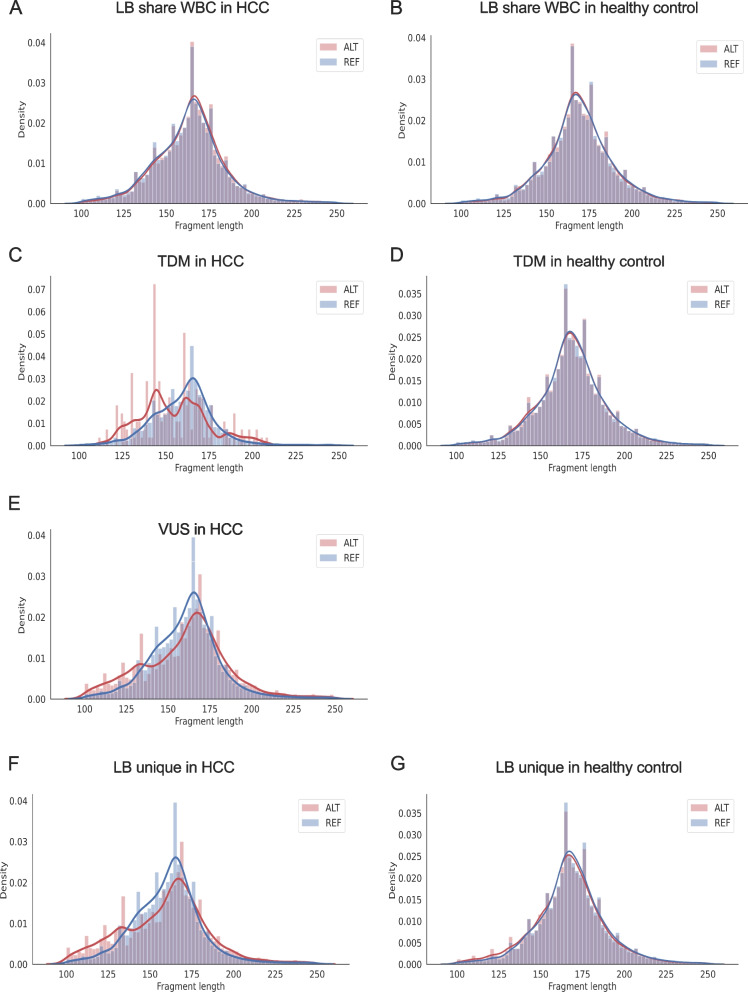
Distinct fragment length patterns of different sources of plasma mutations in PwHCC and healthy individuals. **A** and **B** Distribution of fragment length of sequencing reads carrying WBC-derived mutations (ALT) in PwHCC (**A**) and healthy individuals (**B**), as compared to their corresponding reference reads (REF). **C** and **D** Distribution of fragment lengths of sequencing reads carrying TDMs in PwHCC (**C**) and TDM shared mutations (**D**) detected in healthy participants, as compared to their corresponding reference reads (REF). **E** Distribution of fragment length of sequencing reads carrying variants of unknown sources (VUS) detected in plasma samples of HCC patients. **F** and **G** Distribution of fragment lengths of sequencing reads carrying LB-unique mutations in PwHCC (**F**) and healthy individuals (**G**), as compared to their corresponding reference reads (REF)

We decided to examine whether the fragment length of mutant reads could be exploited to differentiate sources of mutations. We found that the reads bearing LB-share-WBC mutations in both PwHCC and healthy individuals overlapped in fragment length density with their corresponding reference reads (Fig. 3A and B). Interestingly, the fragment length distribution of the TDM-bearing reads in PwHCC was different from LB-share-WBC mutations (Fig. 3C). Specifically, the profile of the TDM reads in 55 PwHCC skewed to the left and peaked at 150 bp, indicating a preponderance of fragments shorter than 150 bp (Fig. 3C), which is consistent with previous studies [40, 41] showing that ctDNA fragments tended to be shorter than other cfDNA fragments. By contrast, the fragment length distribution of reads containing mutations detected in healthy individuals and shared with TDM in PwHCC (TDM in healthy control) overlapped with their reference reads, resembling the profiles of background WDM (Fig. 3D). This suggested that DNA fragments carrying TDMs could be distinguished from non-tumor cfDNA fragments despite harboring overlapping mutations. Although the peak at 150 bp was less obvious, VUS fragments which may consist of not yet confirmed TDMs also displayed left-skewed profiles like that of TDMs (Fig. 3E). The unique signatures of TDM and VUS bearing fragments prompted us to examine whether aggregation of fragment length of all LB-unique mutations detected in plasma after excluding background WDM mutations could distinguish PwHCC from healthy individuals.

Like TMDs and VUS, LB-unique mutations of 55 PwHCC displayed a left skewness in their fragment length distribution compared to their reference reads (Fig. 3F, Fig. S5A). By contrast, the distribution of fragment length of LB-unique mutations of 55 healthy participants overlapped with their reference reads, which was similar to the fragment patterns of WDMs (Fig. 3G, Fig. S5B). Thus, these results suggested that cfDNA fragments containing LB-unique mutations in PwHCC had length distributions distinct from that of healthy participants, which could serve as potential markers for detection of ctDNA.

### Machine learning model built from fragment length profiles of LB-unique mutations could distinguish HCC patients from healthy participants

**Fig. 4:**
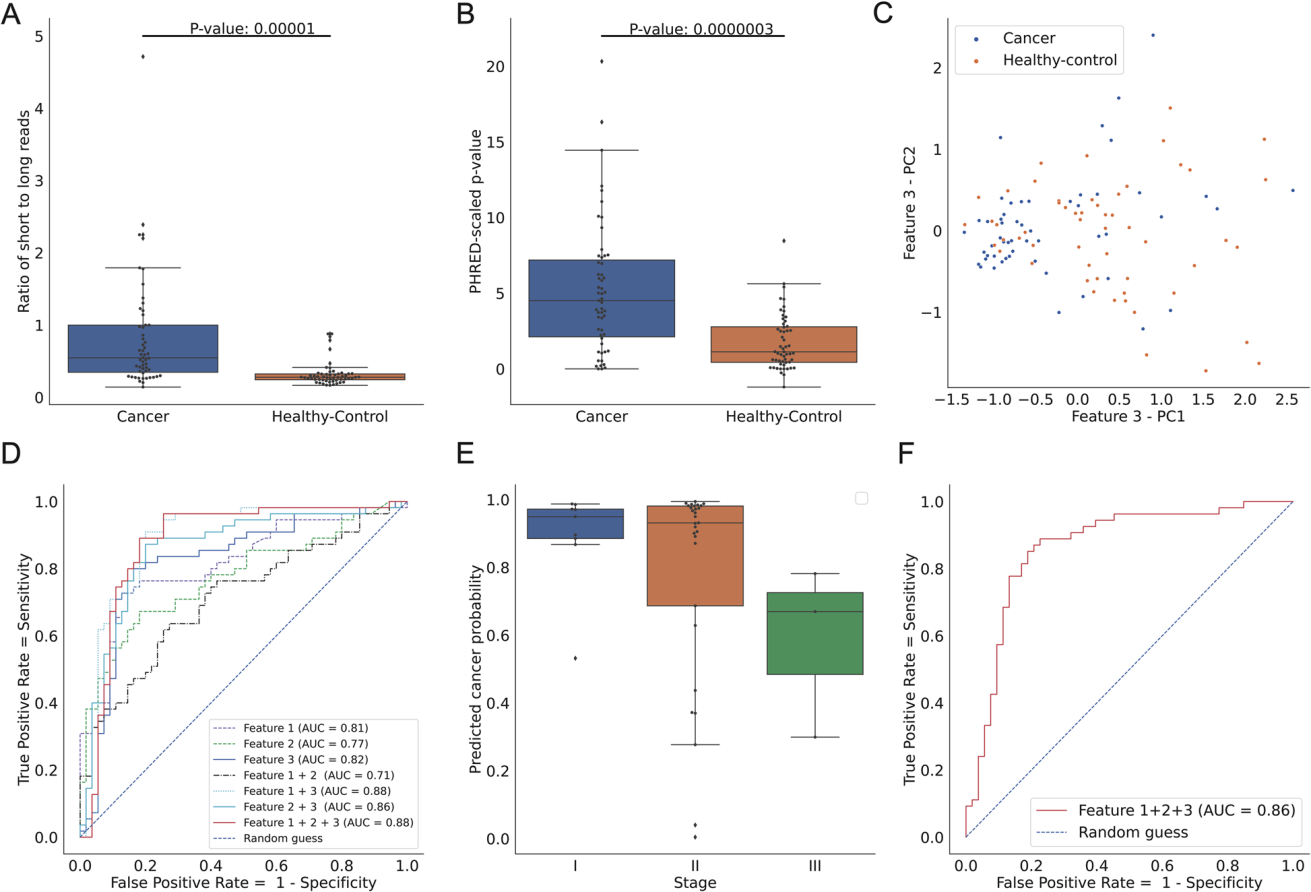
Fragment length profiles of LB-unique mutations could distinguish liquid biopsy samples of patients with early-stage HCC from healthy individuals. **A** Comparison of ratios of short (< 150 bp) to long (> 150 bp) reads bearing LB-unique mutations (feature 1) between PwHCC and healthy individuals in the discovery cohort. Box plots include the median line, *p*-value estimated by the one-tailed Mann–Whitney U test. **B** Comparison of PHRED-scaled *p*-values obtained from enrichment analysis of fragment length distribution in specific ranges (feature 2, Materials and methods sections) of all fragments bearing LB-unique mutations from PwHCC with that of healthy individuals. Box plots include the median line, *p*-value was using the one-tailed Mann–Whitney U test. **C** Principal component analysis of $\lambda \left(s\right)$ over a sliding and non-overlapping window of 10 consecutive values of $s$, $s\in \{100, 101,..., 250\}$, which yields a 15-dimensional feature vector (feature 3) in PwHCC and healthy individuals. **D** ROC curves showing the classification power of individual features and their combinations. **E** Box plot comparing the cancer prediction scores of patients with different tumor stages including stage I (*n* = 9), stage II (n-29) and stage III HCC (*n* = 3). **F** ROC curve showing the performance of models built by combining the three signatures of fragment length of LB-unique mutations in an independent validation cohort. bp: base pair

To further highlight the differences in fragment length profiles between PwHCC and healthy participants, we first compared the ratios of short reads (< = 150 bp) to long reads (> 150 bp) that contained LB-unique mutations between the two groups (feature 1, Fig. S6A). We observed a significant increase (Fig. 4A, *p* = 0.00115) in the fraction of short reads bearing LB-unique mutation in PwHCC compared to healthy participants. By focusing on fragments in a specific length range between 110 and 135 bp (feature 2, Fig. S6B), we observed that the probability of finding such fragments in LB from PwHCC is significantly higher than from healthy participants (Fig. S5B and Fig. 4B, *p* < 0.001). The third fragmentomic feature (feature 3, Fig. S6C) is the probability of detecting reads containing cancer mutations at a particular size s (s ranges from 100 to 250 bp). For this feature, we generated a 15-dimensional vector containing 15 values corresponding to 15 windows of 10 bp as described in the Materials and methods section. The principal component analysis showed that samples taken from PwHCC tended to cluster together while data revealed that samples taken from healthy participants did not form a clear cluster (Fig. 4C). To evaluate the classification performance of these individual feature types and their combinations, we used a gradient-boosting tree-based algorithm and performed leave-one-out cross-validation strategy for all the samples in the discovery cohort. We found that the model built from feature 3 provided the greatest classification power with AUC of 0.82, followed by feature 1 and feature 2 with AUC of 0.81 and 0.77, respectively (Fig. 4D). The combination of all three features achieved the best performance with AUC of 0.88 (Fig. 4D), sensitivity of 89%, and specificity of 82% (Table 4). There were no significant differences in the cancer prediction scores between patients with stage I HCC and those with stage II HCC (Fig. 4E). Patients with stage III HCC tended to have lower prediction scores, however, more samples are required to draw a firm conclusion (Fig. 4E).

**Table 4:** Sensitivity and Specificity of individual features and combination of features. Feature 1, Fraction of short (< 150 bp) to long (< 150 bp) fragments; Feature 2, Size selection enrichment of fragment size distribution at specific ranges; Feature 3, Probability of observing a fragment of a particular size s

**Marker**	**Cut-off**	**Specificity (Discovery)**	**Sensitivity (Discovery)**	**Specificity (Validation)**	**Sensitivity (Validation)**
Feature 1	0.36	84%	75%	83%	96%
Feature 1 & 2	0.60	75%	62%	77%	85%
Feature 1 & 2 & 3	0.37	82%	89%	81%	81%
Feature 1 & 3	0.45	82%	89%	77%	78%
Feature 2	3.36	82%	67%	92%	54%
Feature 2 & 3	0.29	80%	87%	72%	70%
Feature 3	0.47	84%	80%	79%	54%

To comprehensively evaluate the predictive power of the features' abilities to preferentially detect the presence of HCC TDM in LB of PwHCC compared to healthy participants, we tested the combination model on the validation cohort of 54 PwHCC and 53 healthy participants recruited from another hospital (Fig. S1). The model exhibited comparable classification performance with an AUC of 0.86 (Fig. 4F) and yielded a sensitivity and specificity of 81% (Table 4).

Taken together, our results showed that the machine learning model based on fragment length signatures of plasma mutation bearing reads could overcome the challenges of using cancer-specific mutations to discriminate blood samples of patients with early-stage HCC from healthy individuals.

## Discussion

Like many cancers, early detection improves prognosis and survival rates of PwHCC. But current detection methods primarily rely upon imaging and a blood test for a non-specific tumor marker, alpha-fetoprotein, which showed inefficacy in detecting tumors smaller than one centimeter. In the present study, we presented the major challenges of LB assays based on cancer-specific variants and proposed a novel approach combining variant status and their fragment size to overcome challenges and enhance specificity and sensitivity for early detection of HCC.

Consistent with previous studies, we observed that many of the WBC can confound the clear interpretation of TDM in plasma cfDNA [42]. To identify such mutations, we built an in-house probabilistic model using the frequency of altered alleles and total coverage of cfDNA and WBC sequences (see Materials and methods). We observed large proportions of background mutations originated from WBC cells (Fig. 1A). However, after implementing our statistical model, we were able to reduce the proportion of WBC derived mutations, which contributed to the high false positives (Fig. S3B). Thus, our zero-inflated Beta distribution model based on modelling different characteristics of WBC derived mutations including VAF, allele depth (AD) and total read depth (DP) enabled the removal of false positive LB-unique mutations. Consistently, other studies using similar methodology demonstrated better than the traditional mutation selection methods that relies on a pre-defined set of somatic mutations, e.g. COSMIC [43, 44]. The model achieved high concordance rates with the more expensive approach of sequencing both plasma cfDNA and paired WBC gDNA at high depth (Fig. 1D). In addition to a small number of driver mutations, each cancer contains several passenger mutations and the classification of driver from passenger mutations is a challenging task in the field [45]. A study by Salvadores et al. [46] showed that passenger mutations could serve as markers to classify a tumor to a tissue-of-origin, which is clinically important for a multicancer detection blood test.

An important drawback of employing mutations as markers for the development of ctDNA screening tests is the overlap of TDM with non-tumor benign somatic mutations. Indeed, a previous study discovered that cfDNA *TP53*-mutated fragments in 11% of 225 non-cancer controls suggests that circulating mutated fragments among individuals without any diagnosed cancer is common [47]. In agreement with their conclusion, we found 10/55 healthy individuals in the discovery cohort (Fig. S3) carrying mutations overlapping with mutations identified as TDMs, resulting in high detection rates of false-positive mutations. Interestingly, we showed that different sources of mutations could be differentiated by profiling their fragment length patterns (Fig. 3). We observed remarkable differences in the fragment length patterns of tumor-derived mutations (TDM, Fig. 3C) or LB-unique mutations (Fig. 3F) between HCC patients and healthy individuals (Fig. 3D and G). Specifically, the fragment length distribution of TDM or LB-unique mutations in HCC patients represented a nearly bi-modal distribution with a smaller peak at approximately 145–155 bp, while that pattern was not observed for TDM and LB-unique mutations in healthy individuals. Thus, our data demonstrated a unique fragment length signature of mutations detected in plasma of HCC patients which were in line with previous studies reporting that tumor cfDNA fragments tend to be shorter than non-tumor cfDNA fragments [16, 19, 21, 41]. These studies showed that the fragmentation pattern of cfDNA is a non-random event mediated by apoptotic dependent caspases. It has been shown that fragment size distribution of non-tumor cfDNA shows a prominent size of 167 bp corresponding to DNA wrapped around histone (~ 147 bp) plus linker region (~ 10 bp). By contrast, ctDNA fragments have been shown to be around 145 bp [16, 19, 21, 41]. Such size differences are attributed to the differences in nucleosomal organization and chromatin accessibility between non-tumor cfDNA and ctDNA [16]. In support of this notion, ctDNA has been shown to have more accessible chromatin than non-tumor DNA, which may be linked to the highly active transcriptional state of these regions [48]. A recent and remarkable study by Cristiano et al. [19] reported that enrichment for fragments shorter than 150 bp improves the detection of ctDNA. Consistently, we showed that the analysis of fragment length signatures of cancer-specific mutations could be exploited to distinguish HCC patients and healthy controls.

Our combination model interrogating three distinct length signatures of cancer mutation bearing fragments. We examined models built from single feature 1, 2, or 3; combination of two features 1 + 2, 1 + 3, or 2 + 3; and combination of all three. Our data (Fig. 4D and Table 3) showed that the combination of all three features yielded the best performance, suggesting that these features were not redundant. Based on these observations, we further demonstrated that the analysis of three fragment length signatures of aggregated LB-unique mutations in 13 HCC-associated genes could overcome confounding effects of mutation markers and achieved a good AUC of 0.87 for determining the presence of HCC.

The heterogeneity of cancer mutations poses a challenge for using these mutations as markers for the early detection of HCC [49]. Tumor heterogeneity has been reported in HCC at three distinct levels, including interpatient heterogeneity, inter-tumor heterogeneity and intra-tumor heterogeneity [50]. In this study, we showed the interpatient heterogeneity of tumor-derived mutations among 55 HCC patients (Fig. 2) and that by using fragment length signatures of reads bearing plasma mutation rather than mutations themselves, the impact of patient-to-patient variation in their mutational profile could be minimized. However, the other two levels of heterogeneity that represent the differences in mutation profiles between tumor nodules of the patients or between different regions within the same nodule have not been addressed in this study by using a single region sampling strategy. To characterize these aspects of tumor heterogeneity, a multi-region sampling approach has been suggested by several studies [51]. However, the feasibility of this approach is low due to its invasiveness and limited access to tissue samples. Instead, we speculated that our approach based on the integration of fragment length profiles could overcome the intratumor and intertumor heterogeneity of tumor mutations due to its unbiased sampling of ctDNA in the bloodstream and thus might provide a more comprehensive landscape of mutations in HCC patients.

The performance of our assay was comparable to previous studies that developed diagnostic models for early detection of HCC. Jiang et al. showed that quantitative assessment of cfDNA preferred end coordinates and somatic variants allowed researchers to distinguish PwHCC from healthy study participants [18]. Like ours, their assay achieved an area under the ROC of 0.88. However, they evaluated the performance of their model using a fixed cut-off value and have not reported validation using an independent cohort. More recently, HCCseek, another blood-based assay, achieved 75.0% sensitivity at 98.0% specificity [52]. This assay requires shallow whole-genome sequencing of cfDNA to detect copy number variations (CNV) and short fragment lengths, plus the detection of plasma α-fetoprotein. By simultaneous analysis of 5-Hydroxymethylcytosine, end motif, fragment size, and nucleosome footprint profiles of cfDNA, Chan and colleagues could achieve a sensitivity of 95.79% and a specificity of 95.00% for differentiating PwHCC from healthy participants [53]. These studies showed that the performance of LB assays are currently varied across studies and that combining multiple signatures of ctDNA could improve the sensitivity and specificity for early detection of HCC. We assert that combination with other ctDNA biomarkers such as methylated DNA and altered chromosomal copy numbers could increase the accuracy of liquid biopsies and warrant more in-depth study [54]. Thus, future studies are required to test if this multimodal ctDNA analysis would improve our current specificity of 81%, which is an important criterion for an early cancer screening test.

Our study did have a few limitations. The main limitation of our study is the small sample size for each tumor stage group. We attribute this to the strict selection criteria for early-stage and non-metastatic HCC, which is when cancer detection confers significant clinical benefits. Thus, our current study might be considered as exploratory analyses and future studies with a larger cohort are required for robust validation of our assay performance.

Despite being confirmed to have nonmetastatic HCC, tumor-staging and histological records were not available for some HCC patients in the validation cohorts because those patients agreed to participate in the study but later chose to undergo treatment at other hospitals. The design did not include participants without cancer but with known risk factors for HCC, like cirrhosis or HBV.

Our study lacks clinical follow-up with information on the health and disease status of healthy subjects. This is important since a healthy individual may carry cancer-related mutations and subsequently develop cancer. Hence, future case–control studies with larger data sets and follow-up assessments are required to validate the performance of our assay for detection of HCC patients at early stages and to understand the mechanism of tumorigenesis. A recent large-scale pan-cancer analysis of the evolutionary history of tumors by Gerstung et. al [55] has revealed that cancer-causing mutations can occur decades before diagnosis. Thus, investigating the sequence and chronology of mutations leading to cancer will assist in understanding the mechanisms of tumorigenesis as well as offer the possibilities to identify a set of tumor-derived mutations occurred in the precancerous stages for early diagnosis.

Our PwHCC were older, and all our cohorts consisted of a preponderance of men which could be confounding factors of our assay. However, we did not observe any significant association between age or genders with cfDNA fragment length patterns or mutation detection rates (data not shown). A recent study performing genome-wide sequencing of cfDNA and showed elevated amounts of fragments with size smaller than 115 bp in systemic lupus erythematosus patients [56]. Hence the inflammatory condition in such autoimmune diseases might introduce the confounding factor to our analysis that focuses on fragment length patterns of cfDNA fragments. Lastly, although the performance of our model was validated in an independent cohort from a different hospital, the numbers of patients and controls in each cohort were relatively small, thus it would be helpful to test our model in a large prospective clinical study. Future studies could include high-risk patients who are diagnosed with chronic liver diseases such as hepatitis and cirrhosis, to evaluate the ability of our method to distinguish cancer-derived mutations from benign somatic mutations found in those high-risk patients.

## Conclusions

In conclusion, our study provides a novel approach for analyzing cancer-specific mutation status and fragment lengths concomitantly from cfDNA isolated from plasma. Our model includes a probabilistic model to distinguish WDM mutations from ctDNA sequences in plasma by modeling the distributions of VAF occurrences and allele read depth of the two mutation groups. Thus, our study reveals that by assaying the appropriate fragmentomic characteristics of cfDNA while removing the signatures of WBD mutations, it is possible to distinguish, from a blood sample, people with early-stage HCC from healthy people. This strategy of combining cell-free DNA fragment length with multiple characteristics associated with tumor-derived DNA could overcome the limitations of using mutations as sole biomarkers for the detection of ctDNA and improve the accuracy of early screening of HCC.

## Supplementary Information

**Additional file 1: Fig. S1.** Study design chart. The discovery cohort consists of 55 patients with stage I, II and IIIA HCC and 55 healthy volunteers. Ultra-deep sequencing using a panel of the 13 genes found most often mutated among PwHCC were performed on WBC gDNA, FFPE-tumor tissue gDNA and plasma cell-free DNA to identify TDMs and the challenges in classifying the two groups of patients. Mutation fragment length profiles were used as input features to build a machine learning model. The model's performance was subsequently validated in an independent cohort of 55 HCC patients and 53 healthy individuals recruited from a different site. **Figure S2.** Comparison of on target rate and depth coverage between cfDNA and paired WBC gDNA sequencing. (A) and (B) On target rate (A) and mean depth coverage (B) of paired liquid biopsy cfDNA and WBC gDNA samples from 55 HCC patients in the discovery cohort. (C) Bar graph showing the amount of cfDNA (ng per ml) of plasma from HCC and healthy control samples. ns: not significant, Mann–Whitney U test. **Fig. S3.** Tumor-derived mutations in plasma samples of PwHCC overlapped with mutations detected in plasma of healthy participants. (A) Oncoprint plots of distributions of TDMs in 55 healthy individuals. Each row represents a TDM with mutation labeled on the right side and left side shows the occurrences of each mutation. Each column represents a patient. (B) Percentages of mutations shared between liquid biopsies and paired WBCs (LB-share-WBC, blue) and mutations uniquely found in liquid biopsy (LB-unique-variants, orange) for each patient after implementing our statistical model. **Fig. S4.** PwHCC patients displayed cfDNA fragment length profiles distinct from healthy individuals. (A) Length distribution of cfDNA fragments in plasma samples of 55 PwHCC and 55 healthy individuals. (B) The mean ratio of short (≤ 150 bp) to long (> 150bp) fragments across 22 chromosomes at 5Mb resolution for 55 PwHCC and 55 healthy individuals. **Fig. S5.** Distribution of fragment length of sequencing reads carrying LB-unique mutations (ALT) in all 55 PwHCC (A) and 55 healthy individuals (B), as compared to their corresponding reference reads (REF) in the discovery cohort. **Fig. S6.** Graphic explanation of three features generated from analyzing fragment length distribution of LB-unique mutations. (A) Feature 1: The fraction of short-to-long reads that carry LB-unique mutations compared to their corresponding reference reads (RF) in PwHCC (left) and healthy controls (right). (B) Feature 2: Analysis of fragment length distribution of all LB-unique fragments enriched in specific regions (e.g., 110-135 bp). PHRED-scaled *p*-value obtained from Fisher's exact test used to compare the distribution of ALT-fragments in selected regions. (C) Feature 3: The probability of observing an ALT-fragment of size *s* (dot points) in all ALT fragments by proportions was calculated (λ) and the sum of λ(s) over a sliding and non-overlapping window of 10 consecutive values of s was computed to yield a 15-dimensional feature vector.

**Additional file 2: Table S1A.** Clinical characteristics of patients and healthy controls in the discovery cohort. **Table S1B.** Clinical characteristics of patients and healthy controls in the validation cohort. **Table S2.** Gene panel for targeted sequencing. **Table S3.** Frequencies of mutations of difference sources in 55 HCC patients.

## Data Availability

The data presented in this study are available on request from the corresponding author. The data are not publicly available due to ethical regulation.

## Abbreviations

HCC: Hepatocellular Carcinoma
PwHCC: Persons with Hepatocellular Carcinoma
AD: Allele Depth
AUC: Area Under the Curve
cfDNA: Circulating cell-free DNA
ctDNA: Circulating tumor DNA
CNV: Copy Number Variation
DP: Read Depth
FFPE: Formalin-Fixed, Paraffin-Embedded
gDNA: Genomic DNA
HBV: Hepatitis B Virus
LB: Liquid Biopsy
PHRED: Phil's Read Editor
ROC: Receiver Operating Characteristic
SNV: Single Nucleotide Variant
TDM: Tumor-Derived Mutation
UMI: Unique Molecular Identifier
UMT: Unique Molecular Tag
VAF: Variant Allele Fraction
VUS: Variant Of Unknown Source
WBC: White Blood Cell
WDM: White Blood Cell-Derived Mutation
