# Supplementary material for: Fragment length profiles of cancer mutations enhance detection of circulating tumor DNA in patients with early-stage hepatocellular carcinoma
Source: BMC Cancer. 2023 Mar 13;23:233. doi: 10.1186/s12885-023-10681-0 (PMC10009971; doi:10.1186/s12885-023-10681-0)
Supplement: Supplementary file 1 — Additional file 1: Fig. S1. Study design chart. The discovery cohort consists of 55 patients with stage I, II and IIIA HCC and 55 healthy volunteers. Ultra-deep sequencing using a panel of the 13 genes found most often mutated among PwHCC were performed on WBC gDNA, FFPE-tumor tissue gDNA and plasma cell-free DNA to identify TDMs and the challenges in classifying the two groups of patients. Mutation fragment length profiles were used as input features to build a machine learning model. The model's performance was subsequently validated in an independent cohort of 55 HCC patients and 53 healthy individuals recruited from a different site. Figure S2. Comparison of on target rate and depth coverage between cfDNA and paired WBC gDNA sequencing. (A) and (B) On target rate (A) and mean depth coverage (B) of paired liquid biopsy cfDNA and WBC gDNA samples from 55 HCC patients in the discovery cohort. (C) Bar graph showing the amount of cfDNA (ng per ml) of plasma from HCC and healthy control samples. ns: not significant, Mann–Whitney U test. Fig. S3. Tumor-derived mutations in plasma samples of PwHCC overlapped with mutations detected in plasma of healthy participants. (A) Oncoprint plots of distributions of TDMs in 55 healthy individuals. Each row represents a TDM with mutation labeled on the right side and left side shows the occurrences of each mutation. Each column represents a patient. (B) Percentages of mutations shared between liquid biopsies and paired WBCs (LB-share-WBC, blue) and mutations uniquely found in liquid biopsy (LB-unique-variants, orange) for each patient after implementing our statistical model. Fig. S4. PwHCC patients displayed cfDNA fragment length profiles distinct from healthy individuals. (A) Length distribution of cfDNA fragments in plasma samples of 55 PwHCC and 55 healthy individuals. (B) The mean ratio of short (≤ 150 bp) to long (> 150bp) fragments across 22 chromosomes at 5Mb resolution for 55 PwHCC and 55 healthy individuals. Fig. S5. Dist [file 12885_2023_10681_MOESM1_ESM.pdf]

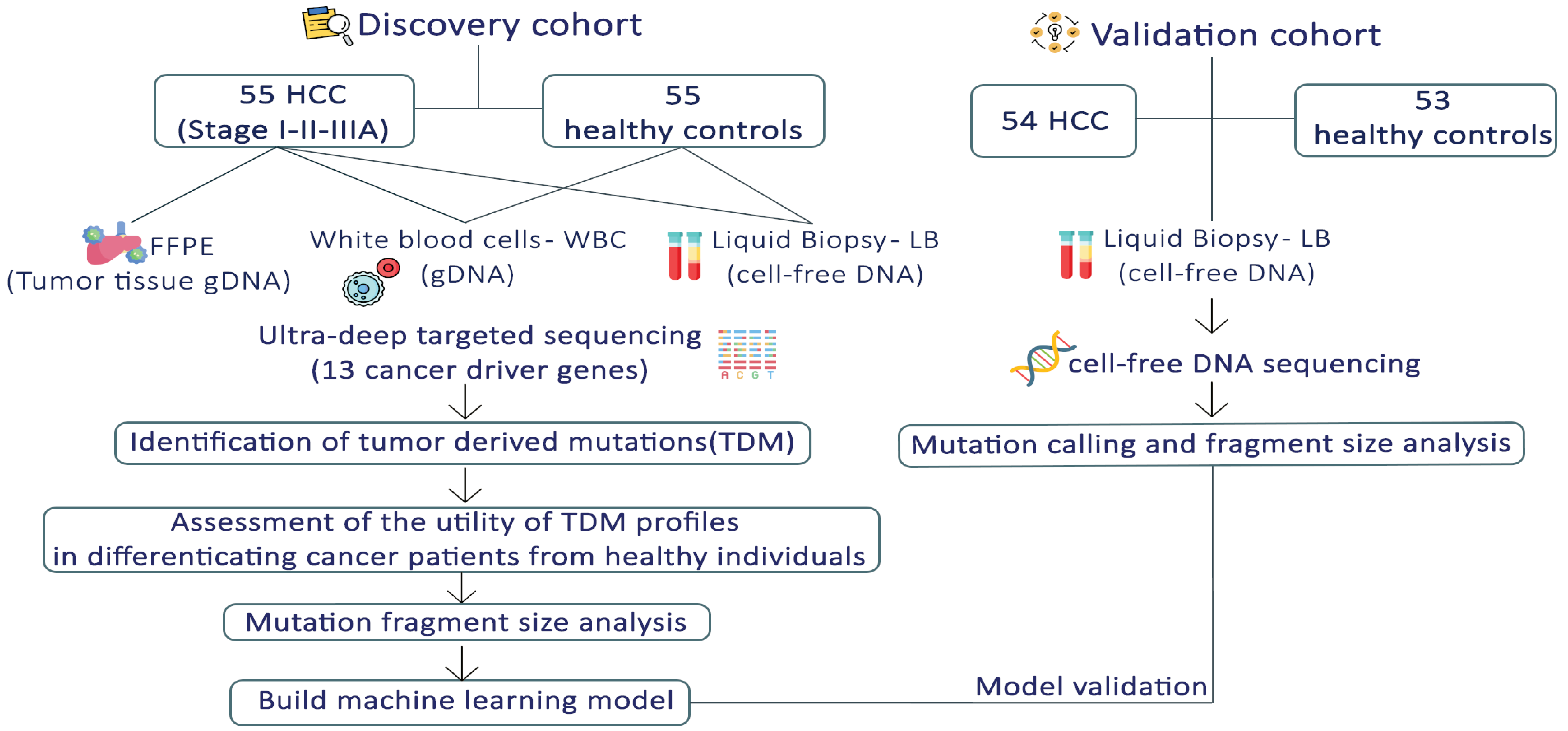
Discovery cohort
55 HCC
(Stage I-II-IIIA)
55
healthy controls
FFPE
(Tumor tissue gDNA)
White blood cells- WBC
(gDNA)
Liquid Biopsy- LB
(cell-free DNA)
Ultra-deep targeted sequencing
(13 cancer driver genes)
Identification of tumor derived mutations(TDM)
Assessment of the utility of TDM profiles
in differentiating cancer patients from healthy individuals
Mutation fragment size analysis
Build machine learning model
Validation cohort
54 HCC
53
healthy controls
Liquid Biopsy- LB
(cell-free DNA)
cell-free DNA sequencing
Mutation calling and fragment size analysis
Model validation

Fig. S1 Study design chart. The discovery cohort consists of 55 patients with stage I, II and IIIA HCC and 55 healthy volunteers. Ultra-deep sequencing using a panel of the 13 genes found most often mutated among PwHCC were performed on WBC gDNA, FFPE-tumor tissue gDNA and plasma cell-free DNA to identify TDMs and the challenges in classifying the two groups of patients. Mutation fragment length profiles were used as input features to build a machine learning model. The model's performance was subsequently validated in an independent cohort of 55 HCC patients and 53 healthy individuals recruited from a different site.

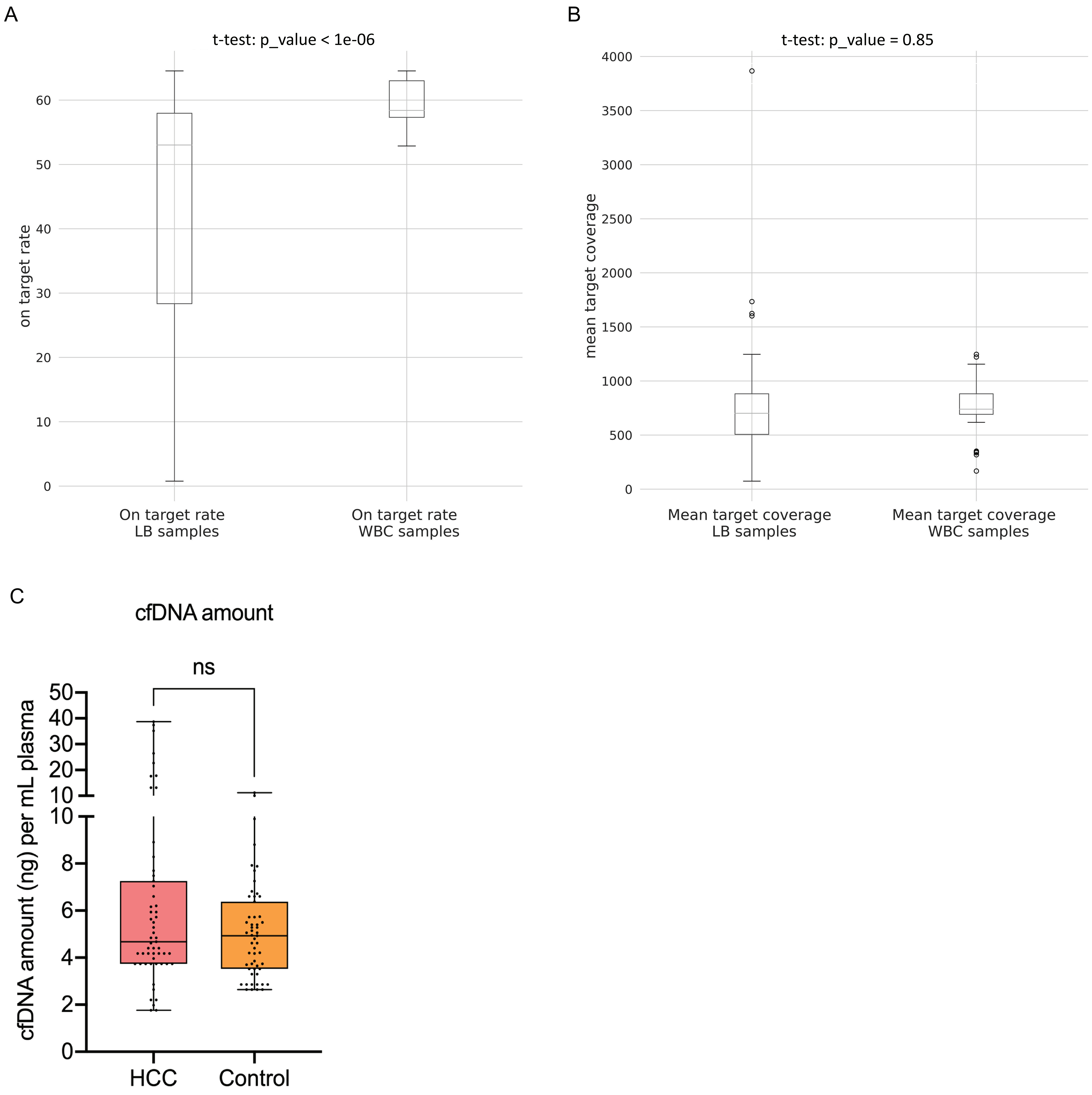
A
t-test: p_value < 1e-06
on target rate
0
10
20
30
40
50
60
On target rate
LB samples
On target rate
WBC samples
B
t-test: p_value = 0.85
mean target coverage
0
500
1000
1500
2000
2500
3000
3500
4000
Mean target coverage
LB samples
Mean target coverage
WBC samples
C
cfDNA amount
ns
cfDNA amount (ng) per mL plasma
0
2
4
6
8
10
10
20
30
40
50
HCC
Control

Figure S2 Comparison of on target rate and depth coverage between cfDNA and paired WBC gDNA sequencing. **(A)** and **(B)** On target rate **(A)** and mean depth coverage **(B)** of paired liquid biopsy cfDNA and WBC gDNA samples from 55 HCC patients in the discovery cohort. (C) Bar graph showing the amount of cfDNA (ng per ml) of plasma from HCC and healthy control samples. ns: not significant, Mann–Whitney U test.

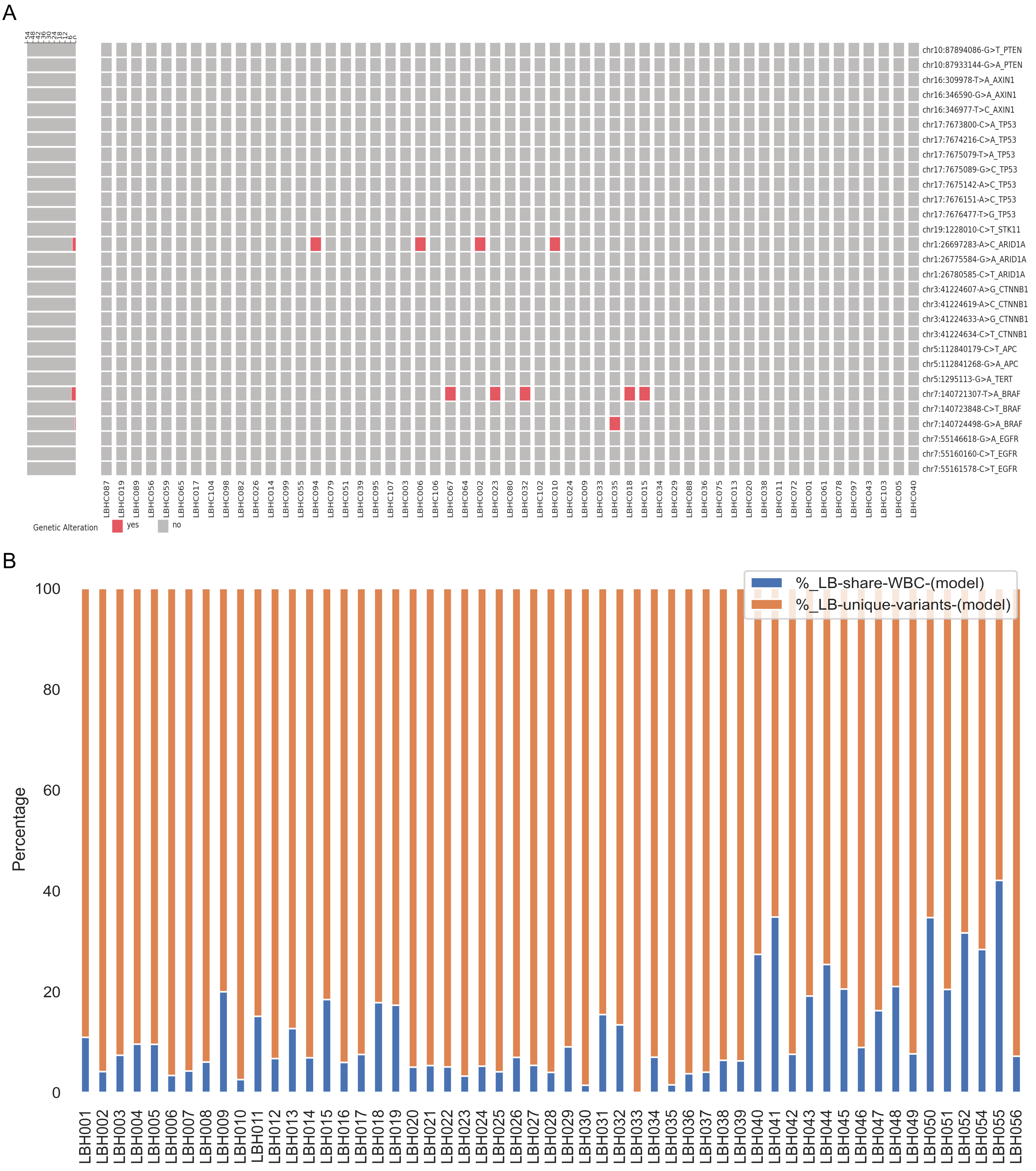

A
chr10:87894086-G>T_PTEN
chr10:87933144-G>A_PTEN
chr16:309978-T>A_AXIN1
chr16:346590-G>A_AXIN1
chr16:346977-T>C_AXIN1
chr17:7673800-C>A_TP53
chr17:7674216-C>A_TP53
chr17:7675079-T>A_TP53
chr17:7675089-G>C_TP53
chr17:7675142-A>C_TP53
chr17:7676151-A>C_TP53
chr17:7676477-T>G_TP53
chr19:1228010-C>T_STK11
chr1:26697283-A>C_ARID1A
chr1:26775584-G>A_ARID1A
chr1:26780585-C>T_ARID1A
chr3:41224607-A>G_CTNNB1
chr3:41224619-A>C_CTNNB1
chr3:41224633-A>G_CTNNB1
chr3:41224634-C>T_CTNNB1
chr5:112840179-C>T_APC
chr5:112841268-G>A_APC
chr5:1295113-G>A_TERT
chr7:140721307-T>A_BRAF
chr7:140723848-C>T_BRAF
chr7:140724498-G>A_BRAF
chr7:55146618-G>A_EGFR
chr7:55160160-C>T_EGFR
chr7:55161578-C>T_EGFR
LBHC087 LBHC019 LBHC089 LBHC056 LBHC059 LBHC065 LBHC017 LBHC104 LBHC098 LBHC082 LBHC026 LBHC014 LBHC099 LBHC055 LBHC094 LBHC079 LBHC051 LBHC039 LBHC095 LBHC107 LBHC003 LBHC006 LBHC106 LBHC067 LBHC064 LBHC002 LBHC023 LBHC080 LBHC032 LBHC102 LBHC010 LBHC024 LBHC009 LBHC033 LBHC035 LBHC018 LBHC015 LBHC034 LBHC029 LBHC088 LBHC036 LBHC075 LBHC013 LBHC020 LBHC038 LBHC011 LBHC072 LBHC001 LBHC061 LBHC078 LBHC097 LBHC043 LBHC103 LBHC005 LBHC040
Genetic Alteration
yes
no
B
%_LB-share-WBC-(model)
%_LB-unique-variants-(model)
Percentage
0
20
40
60
80
100
LBH001 LBH002 LBH003 LBH004 LBH005 LBH006 LBH007 LBH008 LBH009 LBH010 LBH011 LBH012 LBH013 LBH014 LBH015 LBH016 LBH017 LBH018 LBH019 LBH020 LBH021 LBH022 LBH023 LBH024 LBH025 LBH026 LBH027 LBH028 LBH029 LBH030 LBH031 LBH032 LBH033 LBH034 LBH035 LBH036 LBH037 LBH038 LBH039 LBH040 LBH041 LBH042 LBH043 LBH044 LBH045 LBH046 LBH047 LBH048 LBH049 LBH050 LBH051 LBH052 LBH054 LBH055 LBH056

Fig. S3 Tumor-derived mutations in plasma samples of PwHCC overlapped with mutations detected in plasma of healthy participants. (A) Oncoprint plots of distributions of TDMs in 55 healthy individuals. Each row represents a TDM with mutation labeled on the right side and left side shows the occurrences of each mutation. Each column represents a patient. (B) Percentages of mutations shared between liquid biopsies and paired WBCs (LB-share-WBC, blue) and mutations uniquely found in liquid biopsy (LB-unique-variants, orange) for each patient after implementing our statistical model**.**

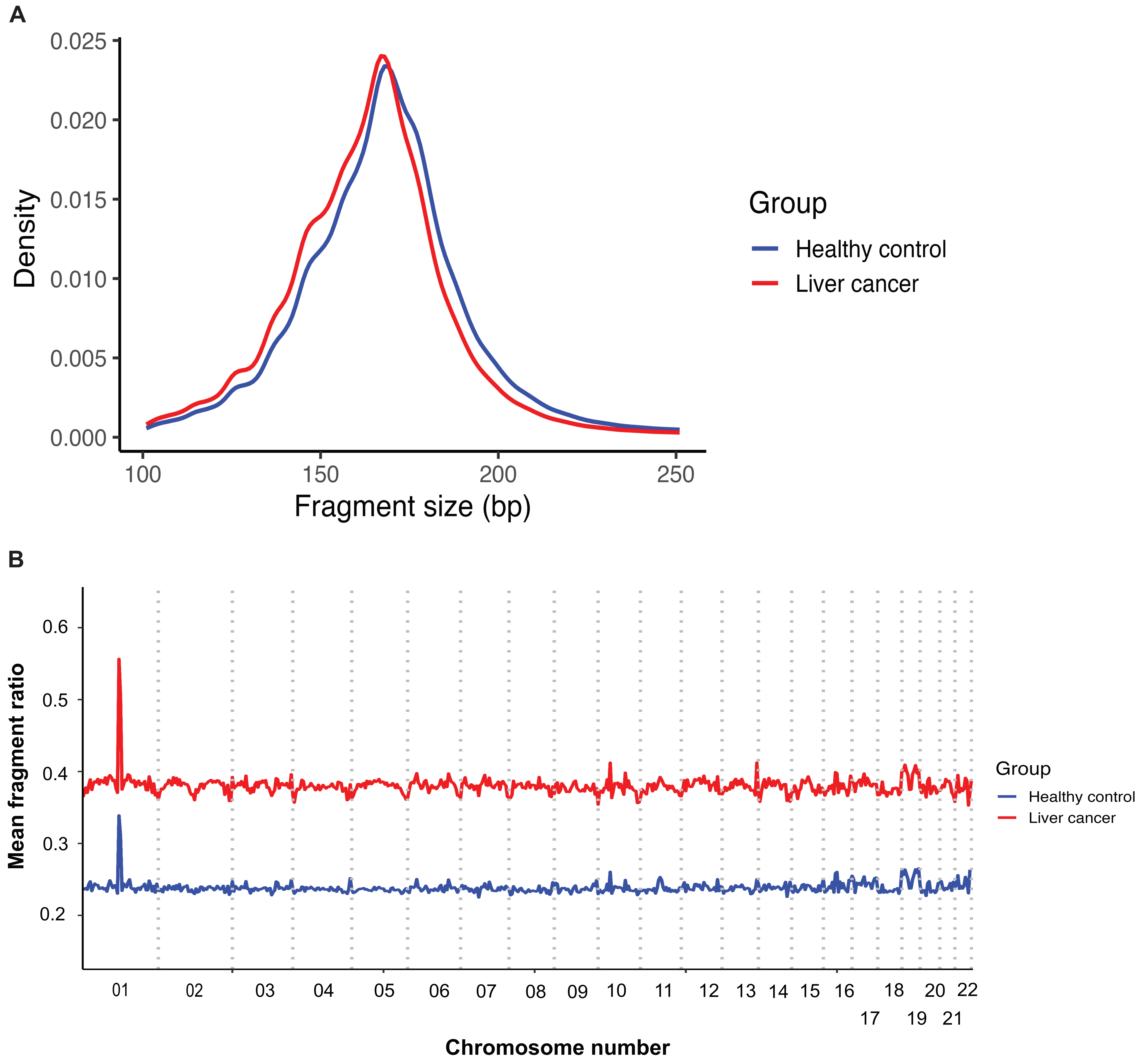
A
0.025
0.020
0.015
0.010
0.005
0.000
Density
100
150
200
250
Fragment size (bp)
Group
Healthy control
Liver cancer
B
0.6
0.5
0.4
0.3
0.2
Mean fragment ratio
01
02
03
04
05
06
07
08
09
10
11
12
13
14
15
16
17
18
19
20
21
22
Chromosome number
Group
Healthy control
Liver cancer

Fig. S4 PwHCC patients displayed cfDNA fragment length profiles distinct from healthy individuals. (A) Length distribution of cfDNA fragments in plasma samples of 55 PwHCC and 55 healthy individuals. **(B)** The mean ratio of short (≤ 150 bp) to long (> 150bp) fragments across 22 chromosomes at 5Mb resolution for 55 PwHCC and 55 healthy individuals.

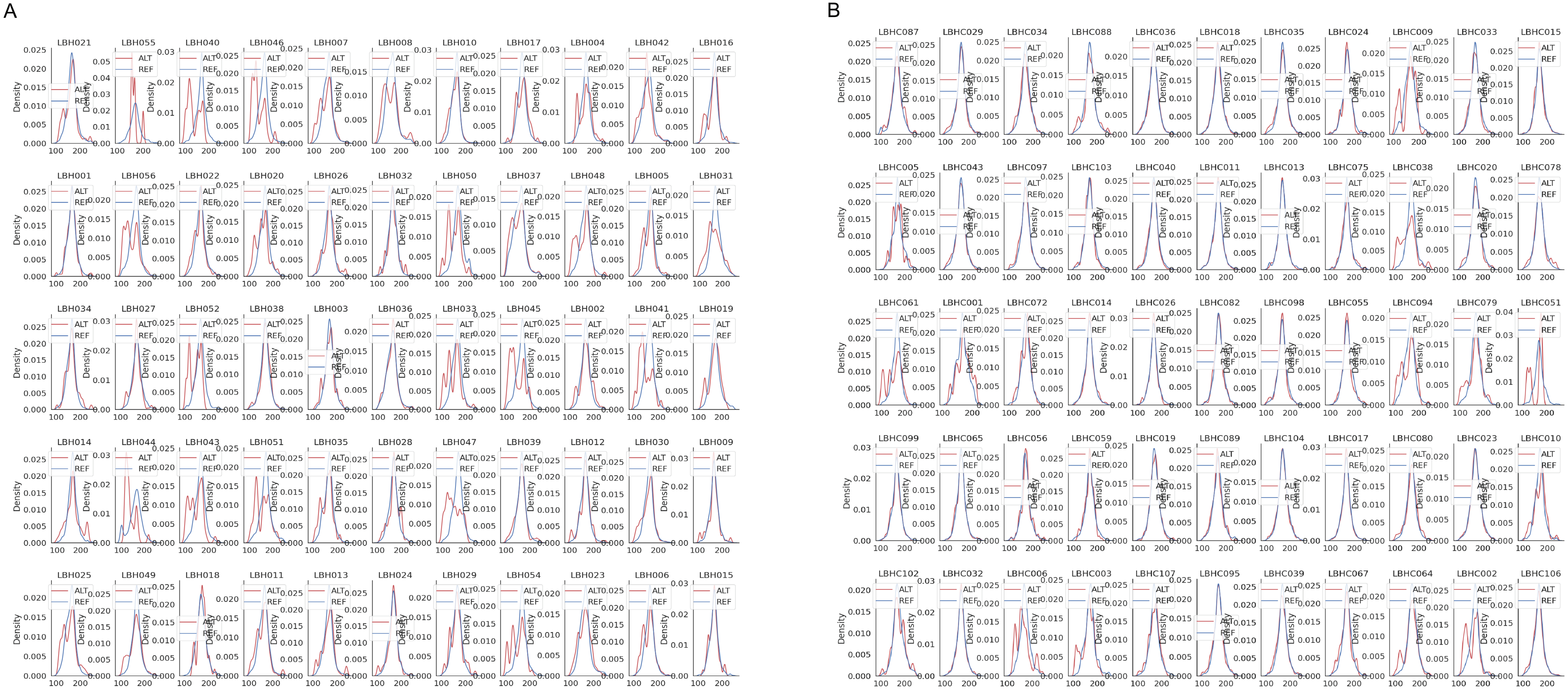
A
LBH021 LBH055 LBH040 LBH046 LBH007 LBH008 LBH010 LBH017 LBH004 LBH042 LBH016
LBH001 LBH056 LBH022 LBH020 LBH026 LBH032 LBH050 LBH037 LBH048 LBH005 LBH031
LBH034 LBH027 LBH052 LBH038 LBH003 LBH036 LBH033 LBH045 LBH002 LBH041 LBH019
LBH014 LBH044 LBH043 LBH051 LBH035 LBH028 LBH047 LBH039 LBH012 LBH030 LBH009
LBH025 LBH049 LBH018 LBH011 LBH013 LBH024 LBH029 LBH054 LBH023 LBH006 LBH015
B
LBHC087 LBHC029 LBHC034 LBHC088 LBHC036 LBHC018 LBHC035 LBHC024 LBHC009 LBHC033 LBHC015
LBHC005 LBHC043 LBHC097 LBHC103 LBHC040 LBHC011 LBHC013 LBHC075 LBHC038 LBHC020 LBHC078
LBHC061 LBHC001 LBHC072 LBHC014 LBHC026 LBHC082 LBHC098 LBHC055 LBHC094 LBHC079 LBHC051
LBHC099 LBHC065 LBHC056 LBHC059 LBHC019 LBHC089 LBHC104 LBHC017 LBHC080 LBHC023 LBHC010
LBHC102 LBHC032 LBHC006 LBHC003 LBHC107 LBHC095 LBHC039 LBHC067 LBHC064 LBHC002 LBHC106
ALT
REF
Density
100 200

Fig. S5 Distribution of fragment length of sequencing reads carrying LB-unique mutations (ALT) in all 55 PwHCC **(A)** and 55 healthy individuals **(B)**, as compared to their corresponding reference reads (REF) in the discovery cohort.

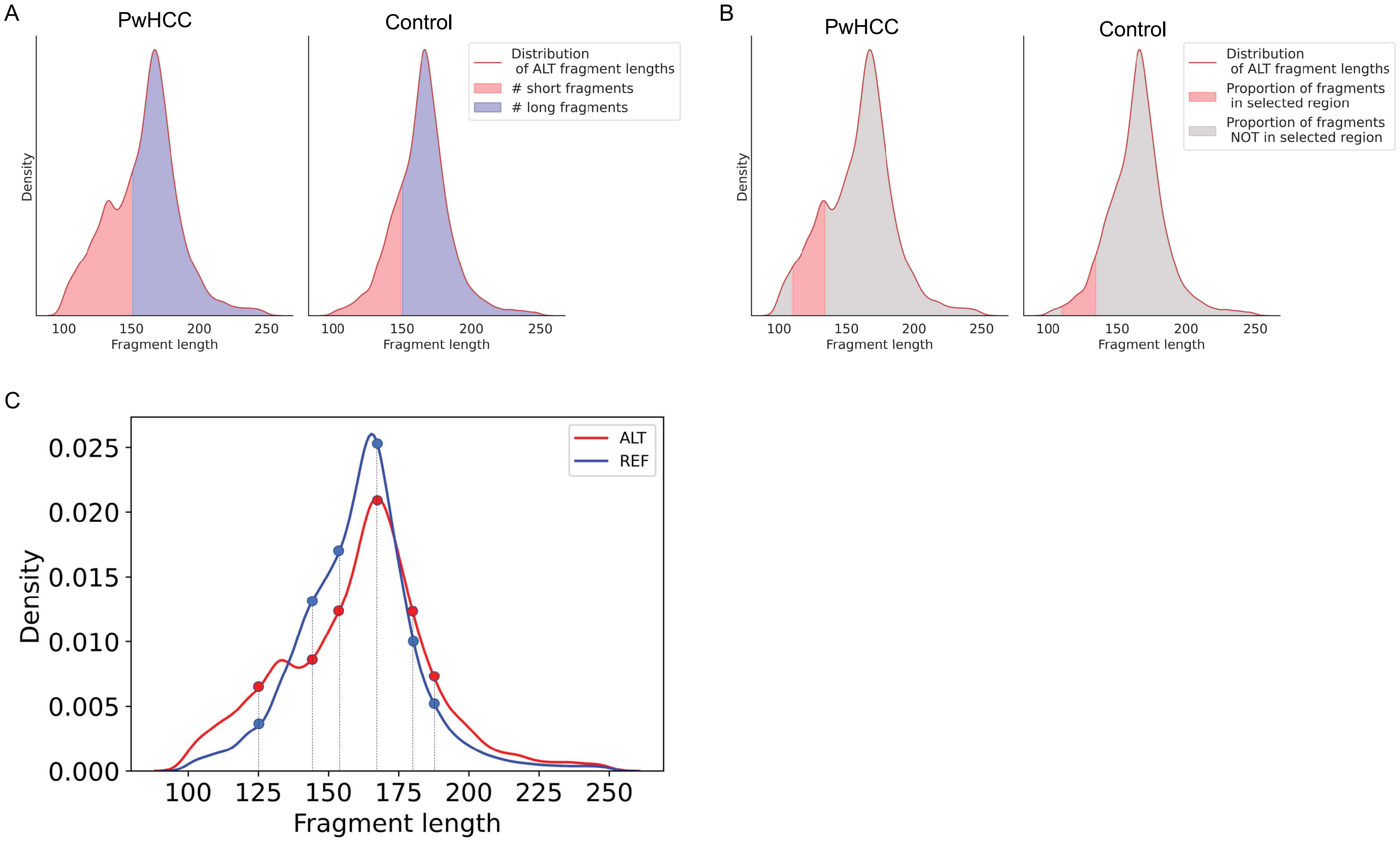
A
PwHCC
Control
Distribution
of ALT fragment lengths
short fragments
long fragments
Density
Fragment length
100
150
200
250
B
PwHCC
Control
Distribution
of ALT fragment lengths
Proportion of fragments
in selected region
Proportion of fragments
NOT in selected region
Density
Fragment length
C
ALT
REF
Density
0.025
0.020
0.015
0.010
0.005
0.000
100
125
150
175
200
225
250
Fragment length

Fig. S6 Graphic explanation of three features generated from analyzing fragment length distribution of LB-unique mutations. **(A)** Feature 1: The fraction of short-to-long reads that carry LB-unique mutations compared to their corresponding reference reads (RF) in PwHCC (left) and healthy controls (right). **(B)** Feature 2: Analysis of fragment length distribution of all LB-unique fragments enriched in specific regions (e.g., 110-135 bp). PHRED-scaled p-value obtained from Fisher's exact test used to compare the distribution of ALT-fragments in selected regions**.** **(C)** Feature 3: The probability of observing an ALT-fragment of size s (dot points) in all ALT fragments by proportions was calculated (λ) and the sum of $\lambda(s)$ over a sliding and non-overlapping window of 10 consecutive values of s was computed to yield a 15-dimensional feature vector.
