# Supplementary material for: Fragment length profiles of cancer mutations enhance detection of circulating tumor DNA in patients with early-stage hepatocellular carcinoma
Source: BMC Cancer. 2023 Mar 13;23:233. doi: 10.1186/s12885-023-10681-0 (PMC10009971; doi:10.1186/s12885-023-10681-0)
Supplement: Supplementary file 2 — Additional file 2: Table S1A. Clinical characteristics of patients and healthy controls in the discovery cohort. Table S1B. Clinical characteristics of patients and healthy controls in the validation cohort. Table S2. Gene panel for targeted sequencing. Table S3. Frequencies of mutations of difference sources in 55 HCC patients. [file 12885_2023_10681_MOESM2_ESM.pdf]

Table S1A: Clinical characteristics of patients and healthy controls in the discovery cohort.

	CODE	Type	Cohort	paired WBC	Age	Gender	Stage	Pathology	Tumor volume	Tumor location	High risk
1	LBH001	Cancer	Discovery	yes	41	M	II	HCC	4 x 4 x 4,5 cm	Seg VI	HBV
2	LBH002	Cancer	Discovery	yes	87	M	I	Carcinoma	2,7 x 4,3 cm	Seg IV	No
3	LBH003	Cancer	Discovery	yes	38	M	NA	NA	NA	NA	HBV
4	LBH004	Cancer	Discovery	yes	36	M	I	HCC	3,5 x 3 x 3 cm	Seg III	HBV
5	LBH005	Cancer	Discovery	yes	57	M	NA	HCC	NA	NA	HBV
6	LBH006	Cancer	Discovery	yes	51	M	II	HCC	3,2 x 3,7 cm	Seg V	HCV
7	LBH007	Cancer	Discovery	yes	58	F	NA	HCC	9,6 x 10,1 cm	NA	NA
8	LBH008	Cancer	Discovery	yes	73	M	I	HCC	13 x 15 x 9 cm	Seg VI-VII	HBV
9	LBH009	Cancer	Discovery	yes	52	M	NA	HCC	3,5 x 3 x 3 cm	Seg III	HBV
10	LBH010	Cancer	Discovery	yes	69	M	NA	HCC	2,7 x 3,6 cm	Seg V	No
11	LBH011	Cancer	Discovery	yes	50	M	I	HCC	9 x 8 x 5 cm	Seg II-III	HBV
12	LBH012	Cancer	Discovery	yes	61	M	NA	HCC	7,5 x 5,5 x 6,5 cm	Right lobe of liver	HBV
13	LBH013	Cancer	Discovery	yes	69	M	NA	HCC	3,2 x 4 x 2,5 cm	Seg VIII	HCV
14	LBH014	Cancer	Discovery	yes	50	M	II	HCC	3,5 x 2,5 x 2,5 cm	Segment	HCV
15	LBH015	Cancer	Discovery	yes	68	M	II	HCC	3,2 x 4 x 2,5 cm	Posterior segments	HBV
16	LBH016	Cancer	Discovery	yes	50	M	I	HCC	3,5 x 2,5 x 2,5 cm	Seg	HBV
17	LBH017	Cancer	Discovery	yes	68	M	I	HCC	93 x 137 x 119 cm	Right liver	HBV

18	LBH018	Cancer	Discovery	yes	51	M	III	HCC	5,8 X 5,4 X 6,2 cm	NA	No
19	LBH019	Cancer	Discovery	yes	35	M	II	Carcinoma	2.5 x 2.5 x 2 cm	Seg VI	HBV
20	LBH020	Cancer	Discovery	yes	75	M	II	HCC	6 x 5 x 5 cm	Anterior segments	HBV
21	LBH021	Cancer	Discovery	yes	64	M	II	HCC	3,5 x 3 x 3 cm	Seg VIII	HBV
22	LBH022	Cancer	Discovery	yes	57	M	III	HCC	7 x 6 x 6 cm	Right lobe of liver	HBV
23	LBH023	Cancer	Discovery	yes	56	M	III	HCC	12 x 8 x 8 cm	Right lobe of liver	HBV
24	LBH024	Cancer	Discovery	yes	71	F	NA	Carcinoma	4,5 x 4 x 3 cm	Left lobe of liver	HBV
25	LBH025	Cancer	Discovery	yes	64	M	II	HCC	2cm	NA	HBV
26	LBH026	Cancer	Discovery	yes	63	M	II	HCC	8 x 5,5 x 7 cm	Left lobe of liver	No
27	LBH027	Cancer	Discovery	yes	28	M	II	HCC	2,5 x 2 x 1,5 cm	Left lobe of liver	No
28	LBH028	Cancer	Discovery	yes	52	M	II	HCC	2,5 x 2,2 x 1,5 cm	Seg IV	HBV/HCV
29	LBH029	Cancer	Discovery	yes	55	M	II	HCC	4 x 3,8 x 3,5 cm	Left liver	HBV
30	LBH030	Cancer	Discovery	yes	67	M	II	HCC	NA	NA	HBV
31	LBH031	Cancer	Discovery	yes	70	M	II	HCC	4,3 x 3,8 x 3 cm	Seg V	HBV
32	LBH032	Cancer	Discovery	yes	56	M	II	HCC	3,5 x 3,2 x 2,8 cm	Anterior segments	HBV
33	LBH033	Cancer	Discovery	yes	50	M	II	HCC	10,5 x 10 x 8 cm	Seg VI	HBV
34	LBH034	Cancer	Discovery	yes	68	F	II	HCC	4,2 x 3,1 x 3,5 cm	Seg VI	HBV

35	LBH035	Cancer	Discovery	yes	59	M	II	HCC	4,6 x 3,2 x 3,5 cm	Left liver	HBV
36	LBH036	Cancer	Discovery	yes	58	M	II	HCC	6,5 x 6 x 5 cm	Left liver	HBV
37	LBH037	Cancer	Discovery	yes	60	M	I	Carcinoma	3,4 x 3 x 3 cm	Seg V	HBV
38	LBH038	Cancer	Discovery	yes	64	F	II	HCC	5,5 x 4 x 3 cm	Posterior segments	HBV
39	LBH039	Cancer	Discovery	yes	74	M	I	Carcinoma	9,8 x 9,5 x 7 cm	Hepatoblastoma	HBV
40	LBH040	Cancer	Discovery	yes	59	M	II	HCC	4 x 3,5 x 2,6 cm	Seg VI	HBV
41	LBH041	Cancer	Discovery	yes	53	M	II	HCC	4,1 x 4,6 cm	Seg V-VI	HBV
42	LBH042	Cancer	Discovery	yes	64	F	NA	HCC	9 x 9 x 7 cm	Right liver	HBV
43	LBH043	Cancer	Discovery	yes	32	M	II	HCC	9,5 x 7,5 x 7 cm	Left liver	HBV
44	LBH044	Cancer	Discovery	yes	24	F	II	HCC	9 x 7 x 6 cm	Seg VI	No
45	LBH045	Cancer	Discovery	yes	36	M	II	HCC	4 x 3 x 3 cm	Seg VIII	HBV
46	LBH046	Cancer	Discovery	yes	77	M	II	HCC	9,5 x 6,5 x 5 cm	Right liver	HBV
47	LBH047	Cancer	Discovery	yes	64	M	NA	HCC	9 x 6 x 5 cm	Right liver	HBV
48	LBH048	Cancer	Discovery	yes	47	M	NA	HCC	7 x 6 x 4,5 cm	Posterior segments	HBV
49	LBH049	Cancer	Discovery	yes	53	M	II	HCC	2 x 1,6 x 1,4 cm	Right liver	HBV
50	LBH050	Cancer	Discovery	yes	59	M	II	HCC	4,5 x 3,2 x 2,8 cm	Seg IV	HBV
51	LBH051	Cancer	Discovery	yes	71	F	NA	HCC	NA	NA	HBV
52	LBH052	Cancer	Discovery	yes	55	M	II	HCC	7,3cm	Left lobe of liver	HBV
53	LBH054	Cancer	Discovery	yes	58	M	I	HCC	4 x 3 x 3.5 cm	Seg IV	HBV

54	LBH055	Cancer	Discovery	yes	36	M	NA	HCC	2.5 x 2 x 2 cm	Posterior segments	HBV
55	LBH056	Cancer	Discovery	yes	61	F	NA	NA	NA	NA	NA
56	LBHC001	Healthy-control	Discovery	yes	43	M	NA	NA	NA	NA	NA
57	LBHC003	Healthy-control	Discovery	yes	27	M	NA	NA	NA	NA	NA
58	LBHC005	Healthy-control	Discovery	yes	28	M	NA	NA	NA	NA	NA
59	LBHC006	Healthy-control	Discovery	yes	35	M	NA	NA	NA	NA	NA
60	LBHC011	Healthy-control	Discovery	yes	34	F	NA	NA	NA	NA	NA
61	LBHC013	Healthy-control	Discovery	yes	61	M	NA	NA	NA	NA	NA
62	LBHC015	Healthy-control	Discovery	yes	48	M	NA	NA	NA	NA	NA
63	LBHC017	Healthy-control	Discovery	yes	44	F	NA	NA	NA	NA	NA
64	LBHC023	Healthy-control	Discovery	yes	61	M	NA	NA	NA	NA	NA
65	LBHC024	Healthy-control	Discovery	yes	37	F	NA	NA	NA	NA	NA
66	LBHC029	Healthy-control	Discovery	yes	50	F	NA	NA	NA	NA	NA
67	LBHC032	Healthy-control	Discovery	yes	69	M	NA	NA	NA	NA	NA
68	LBHC033	Healthy-control	Discovery	yes	62	F	NA	NA	NA	NA	NA
69	LBHC034	Healthy-control	Discovery	yes	43	M	NA	NA	NA	NA	NA

70	LBHC035	Healthy-control	Discovery	yes	48	F	NA	NA	NA	NA	NA
71	LBHC036	Healthy-control	Discovery	yes	31	F	NA	NA	NA	NA	NA
72	LBHC039	Healthy-control	Discovery	yes	42	F	NA	NA	NA	NA	NA
73	LBHC040	Healthy-control	Discovery	yes	40	F	NA	NA	NA	NA	NA
74	LBHC043	Healthy-control	Discovery	yes	36	M	NA	NA	NA	NA	NA
75	LBHC055	Healthy-control	Discovery	yes	28	F	NA	NA	NA	NA	NA
76	LBHC059	Healthy-control	Discovery	yes	81	M	NA	NA	NA	NA	NA
77	LBHC061	Healthy-control	Discovery	yes	40	M	NA	NA	NA	NA	NA
78	LBHC065	Healthy-control	Discovery	yes	61	F	NA	NA	NA	NA	NA
79	LBHC067	Healthy-control	Discovery	yes	37	M	NA	NA	NA	NA	NA
80	LBHC075	Healthy-control	Discovery	yes	37	F	NA	NA	NA	NA	NA
81	LBHC078	Healthy-control	Discovery	yes	41	F	NA	NA	NA	NA	NA
82	LBHC079	Healthy-control	Discovery	yes	46	F	NA	NA	NA	NA	NA
83	LBHC080	Healthy-control	Discovery	yes	70	M	NA	NA	NA	NA	NA
84	LBHC082	Healthy-control	Discovery	yes	49	F	NA	NA	NA	NA	NA
85	LBHC087	Healthy-control	Discovery	yes	55	M	NA	NA	NA	NA	NA

86	LBHC088	Healthy-control	Discovery	yes	37	F	NA	NA	NA	NA	NA
87	LBHC094	Healthy-control	Discovery	yes	25	F	NA	NA	NA	NA	NA
88	LBHC095	Healthy-control	Discovery	yes	48	F	NA	NA	NA	NA	NA
89	LBHC097	Healthy-control	Discovery	yes	46	F	NA	NA	NA	NA	NA
90	LBHC099	Healthy-control	Discovery	yes	47	F	NA	NA	NA	NA	NA
91	LBHC102	Healthy-control	Discovery	yes	39	M	NA	NA	NA	NA	NA
92	LBHC103	Healthy-control	Discovery	yes	67	F	NA	NA	NA	NA	NA
93	LBHC104	Healthy-control	Discovery	yes	52	M	NA	NA	NA	NA	NA
94	LBHC106	Healthy-control	Discovery	yes	47	M	NA	NA	NA	NA	NA
95	LBHC002	Healthy-control	Discovery	yes	29	M	NA	NA	NA	NA	NA
96	LBHC009	Healthy-control	Discovery	yes	34	F	NA	NA	NA	NA	NA
97	LBHC010	Healthy-control	Discovery	yes	38	F	NA	NA	NA	NA	NA
98	LBHC014	Healthy-control	Discovery	yes	60	F	NA	NA	NA	NA	NA
99	LBHC018	Healthy-control	Discovery	yes	61	F	NA	NA	NA	NA	NA
100	LBHC019	Healthy-control	Discovery	yes	41	M	NA	NA	NA	NA	NA
101	LBHC020	Healthy-control	Discovery	yes	37	M	NA	NA	NA	NA	NA

102	LBHC026	Healthy-control	Discovery	yes	40	F	NA	NA	NA	NA	NA
103	LBHC038	Healthy-control	Discovery	yes	30	F	NA	NA	NA	NA	NA
104	LBHC051	Healthy-control	Discovery	yes	39	F	NA	NA	NA	NA	NA
105	LBHC056	Healthy-control	Discovery	yes	35	F	NA	NA	NA	NA	NA
106	LBHC064	Healthy-control	Discovery	yes	35	F	NA	NA	NA	NA	NA
107	LBHC072	Healthy-control	Discovery	yes	40	M	NA	NA	NA	NA	NA
108	LBHC089	Healthy-control	Discovery	yes	48	F	NA	NA	NA	NA	NA
109	LBHC098	Healthy-control	Discovery	yes	44	F	NA	NA	NA	NA	NA
110	LBHC107	Healthy-control	Discovery	yes	43	M	NA	NA	NA	NA	NA

Table S1B: Clinical characteristics of patients and healthy controls in the validation cohort.

	LABCODE	Type	Cohort	Age	Gender	Imaging Diagnosis	Tumor volume	Tumor location	High risk
1	LBHC004	Healthy-control	Validation	35	F	NA	NA	NA	NA
2	LBHC016	Healthy-control	Validation	49	F	NA	NA	NA	NA
3	LBHC021	Healthy-control	Validation	47	F	NA	NA	NA	NA
4	LBHC022	Healthy-control	Validation	17	F	NA	NA	NA	NA
5	LBHC025	Healthy-control	Validation	75	F	NA	NA	NA	NA
6	LBHC027	Healthy-control	Validation	30	M	NA	NA	NA	NA
7	LBHC030	Healthy-control	Validation	42	F	NA	NA	NA	NA
8	LBHC045	Healthy-control	Validation	54	M	NA	NA	NA	NA
9	LBHC048	Healthy-control	Validation	59	M	NA	NA	NA	NA
10	LBHC074	Healthy-control	Validation	28	M	NA	NA	NA	NA
11	LBHC105	Healthy-control	Validation	51	M	NA	NA	NA	NA
12	LBHC007	Healthy-control	Validation	27	M	NA	NA	NA	NA
13	LBHC008	Healthy-control	Validation	48	M	NA	NA	NA	NA
14	LBHC012	Healthy-control	Validation	35	F	NA	NA	NA	NA
15	LBHC028	Healthy-control	Validation	31	F	NA	NA	NA	NA
16	LBHC031	Healthy-control	Validation	53	M	NA	NA	NA	NA
17	LBHC041	Healthy-control	Validation	39	F	NA	NA	NA	NA
18	LBHC047	Healthy-control	Validation	54	M	NA	NA	NA	NA
19	LBHC057	Healthy-control	Validation	35	M	NA	NA	NA	NA
20	LBHC077	Healthy-control	Validation	38	F	NA	NA	NA	NA
21	LBHC037	Healthy-control	Validation	37	F	NA	NA	NA	NA
22	LBHC042	Healthy-control	Validation	28	F	NA	NA	NA	NA
23	LBHC044	Healthy-control	Validation	45	F	NA	NA	NA	NA
24	LBHC046	Healthy-control	Validation	48	F	NA	NA	NA	NA
25	LBHC049	Healthy-control	Validation	42	M	NA	NA	NA	NA
26	LBHC050	Healthy-control	Validation	47	F	NA	NA	NA	NA
27	LBHC052	Healthy-control	Validation	33	F	NA	NA	NA	NA
28	LBHC053	Healthy-control	Validation	61	M	NA	NA	NA	NA

29	LBHC054	Healthy-control	Validation	32	F	NA	NA	NA	NA
30	LBHC058	Healthy-control	Validation	43	M	NA	NA	NA	NA
31	LBHC060	Healthy-control	Validation	45	F	NA	NA	NA	NA
32	LBHC062	Healthy-control	Validation	55	F	NA	NA	NA	NA
33	LBHC063	Healthy-control	Validation	56	M	NA	NA	NA	NA
34	LBHC066	Healthy-control	Validation	46	F	NA	NA	NA	NA
35	LBHC068	Healthy-control	Validation	31	F	NA	NA	NA	NA
36	LBHC069	Healthy-control	Validation	43	M	NA	NA	NA	NA
37	LBHC070	Healthy-control	Validation	26	M	NA	NA	NA	NA
38	LBHC071	Healthy-control	Validation	35	F	NA	NA	NA	NA
39	LBHC073	Healthy-control	Validation	33	F	NA	NA	NA	NA
40	LBHC076	Healthy-control	Validation	48	M	NA	NA	NA	NA
41	LBHC081	Healthy-control	Validation	47	F	NA	NA	NA	NA
42	LBHC083	Healthy-control	Validation	63	F	NA	NA	NA	NA
43	LBHC084	Healthy-control	Validation	64	M	NA	NA	NA	NA
44	LBHC085	Healthy-control	Validation	33	F	NA	NA	NA	NA
45	LBHC086	Healthy-control	Validation	39	M	NA	NA	NA	NA
46	LBHC090	Healthy-control	Validation	40	F	NA	NA	NA	NA
47	LBHC091	Healthy-control	Validation	53	M	NA	NA	NA	NA
48	LBHC092	Healthy-control	Validation	67	M	NA	NA	NA	NA
49	LBHC093	Healthy-control	Validation	58	F	NA	NA	NA	NA
50	LBHC096	Healthy-control	Validation	38	F	NA	NA	NA	NA
51	LBHC100	Healthy-control	Validation	29	M	NA	NA	NA	NA
52	LBHC101	Healthy-control	Validation	47	M	NA	NA	NA	NA
53	LBHC108	Healthy-control	Validation	31	M	NA	NA	NA	NA
54	LBM005	Cancer	Validation	58	M	Non-metastatic HCC	NA	NA	HCV
55	LBM006	Cancer	Validation	73	M	Non-metastatic HCC	NA	NA	HCV
56	LBM010	Cancer	Validation	47	M	Non-metastatic HCC	NA	NA	HBV
57	LBM016	Cancer	Validation	33	M	Non-metastatic HCC	NA	NA	HBV
58	LBM018	Cancer	Validation	63	M	Non-metastatic HCC	NA	NA	HBV
59	LBM022	Cancer	Validation	69	M	Non-metastatic HCC	NA	NA	HCV

60	LBM023	Cancer	Validation	66	M	Non-metastatic HCC	NA	NA	HBV
61	LBM025	Cancer	Validation	69	F	Non-metastatic HCC	NA	NA	HBV
62	LBM028	Cancer	Validation	77	F	Non-metastatic HCC	NA	NA	HBV
63	LBM031	Cancer	Validation	52	M	Non-metastatic HCC	NA	NA	HBV
64	LBM033	Cancer	Validation	56	M	Non-metastatic HCC	NA	NA	HBV
65	LBM036	Cancer	Validation	62	F	Non-metastatic HCC	NA	NA	HCV
66	LBM037	Cancer	Validation	48	M	Non-metastatic HCC	NA	NA	HBV
67	LBM038	Cancer	Validation	73	M	Non-metastatic HCC	NA	NA	NA
68	LBM040	Cancer	Validation	60	F	Non-metastatic HCC	NA	NA	HBV
69	LBM043	Cancer	Validation	65	M	Non-metastatic HCC	NA	NA	HCV
70	LBM053	Cancer	Validation	69	M	Non-metastatic HCC	NA	NA	HBV
71	LBM054	Cancer	Validation	55	M	Non-metastatic HCC	NA	NA	HCV
72	LBM056	Cancer	Validation	64	F	Non-metastatic HCC	NA	NA	HCV
73	LBM065	Cancer	Validation	66	M	Non-metastatic HCC	NA	NA	HCV
74	LBM070	Cancer	Validation	71	M	Non-metastatic HCC	NA	NA	HBV
75	LBM001	Cancer	Validation	72	F	Non-metastatic HCC	NA	NA	No
76	LBM002	Cancer	Validation	51	M	Non-metastatic HCC	NA	NA	No
77	LBM003	Cancer	Validation	34	M	Non-metastatic HCC	NA	NA	HBV
78	LBM004	Cancer	Validation	63	F	Non-metastatic HCC	NA	NA	HBV
79	LBM007	Cancer	Validation	61	F	Non-metastatic HCC	NA	NA	HBV
80	LBM009	Cancer	Validation	71	M	Non-metastatic HCC	NA	NA	HBV
81	LBM011	Cancer	Validation	56	F	Non-metastatic HCC	NA	NA	HBV
82	LBM012	Cancer	Validation	52	M	Non-metastatic HCC	NA	NA	HBV
83	LBM013	Cancer	Validation	57	M	Non-metastatic HCC	NA	NA	HBV
84	LBM014	Cancer	Validation	56	M	Non-metastatic HCC	NA	NA	No
85	LBM015	Cancer	Validation	50	M	Non-metastatic HCC	NA	NA	HBV
86	LBM017	Cancer	Validation	57	M	Non-metastatic HCC	NA	NA	No
87	LBM019	Cancer	Validation	60	M	Non-metastatic HCC	NA	NA	HBV
88	LBM021	Cancer	Validation	61	F	Non-metastatic HCC	NA	NA	HBV
89	LBM024	Cancer	Validation	65	M	Non-metastatic HCC	NA	NA	NA
90	LBM026	Cancer	Validation	64	F	Non-metastatic HCC	NA	NA	HBV

91	LBM027	Cancer	Validation	58	M	Non-metastatic HCC	NA	NA	No
92	LBM029	Cancer	Validation	51	M	Non-metastatic HCC	NA	NA	HBV
93	LBM030	Cancer	Validation	67	F	Non-metastatic HCC	NA	NA	HBV
94	LBM032	Cancer	Validation	51	M	Non-metastatic HCC	NA	NA	HBV
95	LBM034	Cancer	Validation	59	F	Non-metastatic HCC	NA	NA	No
96	LBM035	Cancer	Validation	48	M	Non-metastatic HCC	NA	NA	HBV
97	LBM039	Cancer	Validation	68	M	Non-metastatic HCC	NA	NA	HBV
98	LBM042	Cancer	Validation	46	M	Non-metastatic HCC	NA	NA	NA
99	LBM044	Cancer	Validation	53	M	Non-metastatic HCC	NA	NA	NA
100	LBM045	Cancer	Validation	42	M	Non-metastatic HCC	NA	NA	HBV
101	LBM046	Cancer	Validation	57	M	Non-metastatic HCC	NA	NA	HBV/HCV
102	LBM055	Cancer	Validation	72	F	Non-metastatic HCC	NA	NA	No
103	LBM057	Cancer	Validation	50	M	Non-metastatic HCC	NA	NA	HBV
104	LBM058	Cancer	Validation	72	F	Non-metastatic HCC	NA	NA	HCV
105	LBM069	Cancer	Validation	63	M	Non-metastatic HCC	NA	NA	HCV
106	LBM073	Cancer	Validation	73	F	Non-metastatic HCC	NA	NA	HBV
107	LBM083	Cancer	Validation	55	M	Non-metastatic HCC	NA	NA	HBV

Table S2: Gene panel for targeted sequencing

Target	Target region size (bp)	Chromosome	Start	Stop	Strand
APC	8931	5	112043415	112179823	+
ARID1A	6858	1	27022895	27107247	+
AXIN1	2886	16	338122	402434	-
BRAF	10204	7	140419134	140624305	-
CDKN2A	2627	9	21968228	21994357	-
CTNNB1	2346	3	41265560	41280833	+
EGFR	4952	7	55086971	55273310	+
KRAS	1273	12	25362445	25398329	-
PIK3CA	3207	3	178916614	178952152	+
PTEN	1212	10	89624226	89725229	+
STK11	4617	19	1206913	1228081	+
TP53	1689	17	7572927	7579912	-
TERT (promoter)	300	5	1253282	1295184	-

Table S3 Frequencies of mutations of difference sources in 55 HCC patients

Gene	LB_unique				TDM				LB-shared-WBC				VUS			
	Mutation (N=3620)		Sample (N=55)		Mutation (N=30)		Sample (N=55)		Mutation (N=66)		Sample (N=55)		Mutation (N=3596)		Sample (N=55)	
	Number	Percentage	Number	Percentage	Number	Percentage	Number	Percentage	Number	Percentage	Number	Percentage	Number	Percentage	Number	Percentage
APC	592	16%	53	96%	2	7%	1	2%	7	11%	32	58%	591	16%	53	96%
ARID1A	590	16%	54	98%	3	10%	3	5%	7	11%	13	24%	588	16%	54	98%
AXIN1	259	7%	54	98%	3	10%	3	5%	5	8%	17	31%	257	7%	54	98%
BRAF	621	17%	55	100%	3	10%	3	5%	6	9%	27	49%	619	17%	55	100%
CDKN2A	139	4%	50	91%	0	0%	0	0%	3	5%	27	49%	139	4%	50	91%
CTNNB1	99	3%	30	55%	4	13%	4	7%	1	2%	7	13%	95	3%	28	51%
EGFR	436	12%	49	89%	3	10%	3	5%	13	20%	45	82%	433	12%	49	89%
KRAS	45	1%	22	40%	0	0%	0	0%	3	5%	12	22%	45	1%	22	40%
PIK3CA	162	4%	39	71%	0	0%	0	0%	0	0%	0	0%	162	5%	39	71%
PTEN	93	3%	33	60%	2	7%	2	4%	1	2%	1	2%	91	3%	32	58%
STK11	428	12%	53	96%	2	7%	2	4%	16	24%	32	58%	426	12%	53	96%
TERT	22	1%	25	45%	1	3%	4	7%	1	2%	11	20%	21	1%	23	42%
TP53	134	4%	44	80%	7	23%	11	20%	3	5%	26	47%	129	4%	41	75%
